# Methylation signature of lymph node metastases in breast cancer patients

**DOI:** 10.1186/1471-2407-12-244

**Published:** 2012-06-13

**Authors:** Zeinab Barekati, Ramin Radpour, Qing Lu, Johannes Bitzer, Hong Zheng, Paolo Toniolo, Per Lenner, Xiao Yan Zhong

**Affiliations:** 1 Laboratory for Gynecological Oncology, Women’s Hospital/Department of Biomedicine, University of Basel, Hebelstrasse 20, CH 4031, Basel, Switzerland; 2 Department of Breast Surgery, West China Hospital/West China School of Medicine, Sichuan University, Chengdu, China; 3 Department of Obstetrics and Gynecology, Women’s Hospital, University of Basel, Schanzenstrasse 46, CH-4031, Basel, Switzerland; 4 Department of Oncology, State Key Laboratory of Biotherapy and Cancer Center, West China Hospital/West China School of Medicine, Sichuan University, Chengdu, China; 5 Laboratory of Molecular Diagnosis of Cancer, West China Hospital/West China School of Medicine, Sichuan University, Chengdu, China; 6 Department of Obstetrics & Gynecology, New York University School of Medicine / Institute Universitaire de Médecine Sociale et Preventive, CHUV, Rue du Bugnon 17, 1005, Lausanne, Switzerland; 7 Department of Oncology, Umeå University Hospital, Campus Area, S-90185, Umeå, Sweden

**Keywords:** Methylation, Metastasis, Breast cancer, Biomarker

## Abstract

**Background:**

Invasion and metastasis are two important hallmarks of malignant tumors caused by complex genetic and epigenetic alterations. The present study investigated the contribution of aberrant methylation profiles of cancer related genes, *APC, BIN1, BMP6, BRCA1, CST6, ESR-b, GSTP1, P14 (ARF), P16 (CDKN2A), P21 (CDKN1A), PTEN,* and *TIMP3*, in the matched axillary lymph node metastasis in comparison to the primary tumor tissue and the adjacent normal tissue from the same breast cancer patients to identify the potential of candidate genes methylation as metastatic markers.

**Methods:**

The quantitative methylation analysis was performed using the SEQUENOM’s EpiTYPER™ assay which relies on matrix-assisted laser desorption/ionization time-of-flight mass spectrometry (MALDI-TOF MS).

**Results:**

The quantitative DNA methylation analysis of the candidate genes showed higher methylation proportion in the primary tumor tissue than that of the matched normal tissue and the differences were significant for the *APC, BIN1, BMP6, BRCA1, CST6, ESR-b, P16, PTEN *and *TIMP3* promoter regions (*P*<0.05). Among those candidate methylated genes, *APC, BMP6, BRCA1* and *P16* displayed higher methylation proportion in the matched lymph node metastasis than that found in the normal tissue (*P*<0.05). The pathway analysis revealed that *BMP6, BRCA1* and *P16* have a role in prevention of neoplasm metastasis.

**Conclusions:**

The results of the present study showed methylation heterogeneity between primary tumors and metastatic lesion. The contribution of aberrant methylation alterations of *BMP6, BRCA1* and *P16* genes in lymph node metastasis might provide a further clue to establish useful biomarkers for screening metastasis.

## Background

Breast cancer is one of the most common malignancies with a high mortality rate among women [1]. Breast cancer, a heterogeneous disease, presents various pathological signs such as axillary lymph node metastasis which is associated with a high risk of recurrence and considered as an important prognosis factor in the early stages of the disease [2,3]. Invasion and metastasis are two important hallmarks of malignant tumors associated with complex genetic and epigenetic alterations that allow tumors to disseminate throughout lymphatics or blood vessels, giving rise to the colonization and growth of metastatic cells in distant organs [4-6]. Considering that tumor dissemination is an early event in breast cancer [7], genetic and epigenetic analysis of tumors and metastatic lesions could provide results for biomarker discovery and may improve diagnosis, prognosis and proper management of the treatment for breast cancer patients.

The contribution of aberrant DNA hypermethylation of cancer related genes to the transcriptional silencing and carcinogenesis has been demonstrated in different diseases including different cancer types [7,8]. The methylation profile of genes involved in critical molecular processes such as cell cycle control, DNA repair and angiogenesis in breast cancer has been investigated [9-12]. Since the lymphatic system has a direct dispatch to spread primary tumor cells to the lymph nodes in breast cancer, primary tumor signatures has been considered as surrogate for lymph node metastasis. However, this persuasion has recently been controversial especially in the context of DNA methylation pattern from primary to metastasis in breast cancer [9,10,13]. According to best of our knowledge genome-wide DNA methylation was reported in few metastatic breast cancer cell lines ,however, methylation pattern of individual candidate genes in both the primary and lymph node metastases has been explored in clinical specimens [14,15]. Therefore the profile of metastasis in breast cancer patients is less characterized and poorly understood which needs further studies to understand the relationship between epigenetic alteration and metastases in breast cancer.

Our previous approach revealed methylation signatures of 42, 528 CpG sites from 22 breast cancer candidate genes that demonstrated promoter hypermethylation of 10 genes involved in cell cycle and DNA repair, invasion and metastasis, cell proliferation, signal transduction and cell detoxification [16]. Moreover, we have shown hypermethylation of the two additional genes involved in *TP53* regulatory pathway in the breast cancer patients [17,18]. The significance of using these hypermethylated genes as circulating biomarkers has been explored as well [18]. The present study investigated the contribution of significant aberrant methylation profile of twelve cancer related genes from the aforementioned studies (*APC, BIN, BMP6, BRCA1, CST6, ESR-b, GSTP1, P14, P16, P21, PTEN* and *TIMP3)* in matched axillary lymph node metastasis in comparison to the primary tumor tissue and the adjacent normal tissue from the same breast cancer patients to identify the potential of aberrant methylation profile of the candidate genes as metastatic signature.

## Methods

### Sampling and pathological classification

The study was approved by the local institutional review board (Ethic commission beider Basel, Sichuan University China). Written consent forms were collected from all patients who were involved in this study. Staging and grading was evaluated according to the WHO histological classification. DNA was isolated from 65 samples including matched primary tumors tissue, matched normal tissue and their matched lymph node metastasis of 24 chines patients with breast cancer. The present cohort included 17 matched normal breast tissues that were collected at least 4 cm away from the tumor site and were confirmed as normal tissue by pathologist. The axillary lymph nodes were removed at the same surgery. Part of the samples was embedded using OCT (Optimal Cutting Temperature, Sakura Finetek, U.S.A) and stored in liquid nitrogen. The above procedures were completed within 20 minutes after peeling preparation. The samples were then stored at -80^o^. The frozen tissues were sectioned in 4 μm thickness and were submitted for hematotoxylin and eosin staining study. According to pathological tumor type and immunohistochemistry staining, studied cohort consisted of patient’s with Invasive Ductal Carcinoma (IDC) and Invasive Lobular Carcinoma (ILC). Breast cancer characteristics, such as staging, histological grading, and hormone receptor expression from the breast cancer patients are listed in Table 1.

**Table 1:** Clinical characteristics of the studied cohort

**Total no. of patients**	**Breast cancer tumor type**		**Age mean ± S.D. (range)**	**Pathologic stage**		**No. of patients with distance metastasis**	**Histological grade**			**ER positive patients**	**PR positive patients**	**C-ERB2 positive patients**
	**IDC**	**ILC**		**Early (I,II)**	**Late (III)**		**1**	**2**	**3**			
24	20	4	48 ± 9.53 (33-69)	14	10	0	1	5	18	19	12	12

IDC, Invasive Ductal Carcinoma; ILC, Invasive Lubular Carcinoma; ER, Estrogen Receptor; PR, Progestron Receptor.

The entirely neoplastic and adjacent normal frozen sections were subjected for DNA extraction. Five to ten sections with 90% neoplastic coverage applied for DNA extraction, and last section was checked for the correct characterization, using the High Pure PCR Template Preparation Kit (Roche, Germany). The rest of the samples were fixed in 10% buffered formalin for immunohistochemical staining study.

**Figure 1:**
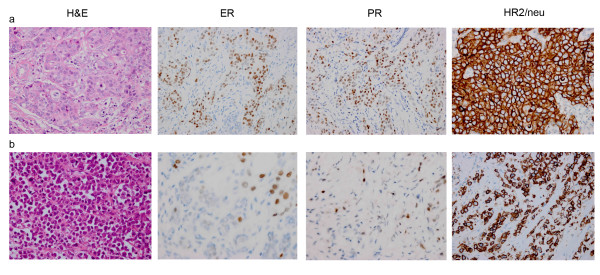
** Hematotoxylin and eosin staining (H&E) and immunohistochemical staining for estrogen receptor (ER), progesterone receptor (PR) and HER2/neu proteins (400X).(a)** Ductal carcinoma. **(b)** Lobular carcinoma.

The analysis for the detection of estrogen receptor (ER), progesterone receptor (PR) and HER2/neu (C-ErbB-2) proteins was carried out using primary antibodies for Rabbit anti-human estrogen receptor monoclonal antibody (clone sp1, Roche), Rabbit anti-human progesterone receptor monoclonal antibody (clone sp2, Roche), Rabbit anti-human HER-2/neu monoclonal antibody (clone 4B5, Roche). Then slide stainings were processed using ultraView Universal DAB Detection Kit (Ventana Medical Systems Inc, Tucson, AZ) (Figure 1). The ER, PR and HER-2/neu were analyzed following ASCO/CAP Guidelines.

### Methylation analysis using thymidine-specific cleavage mass array on MALDI-TOF *silico-chip*

The SEQUENOM’s EpiTYPER™ assay is a methylation quantification method which relies on MALDI-TOF MS [19]. The theory and practice of this approach for DNA methylation quantification has been confirmed by previous studies [16,20,21]. In the current study, this assay was used to quantify the methylation of *APC, BIN1, BRCA1, BMP6, CST6, ESR-b, GSTP1, P14, P16, P21, PTEN* and *TIMP3* promoter regions.

#### Bisulfite treatment

Bisulfite conversion of the target sequences was performed according to the instruction of the Epitect® Bisulfite Kit (QIAGEN AG, Basel, Switzerland).

#### Primer designing and PCR-tagging for EpiTYPER^TM^ assay

To design PCR primer for the candidate genes, CpG density and CpG sites of the twelve targeted sequences were analyzed. According to our previous publications, we used the same primer sequences which were tagged with T7-promoter for the reverse and a 10 mer sequences to the forward primer to balance the PCR condition and primer pairs can cover the promoter regions with the most CpG sites using MethPrimer. The primer sequences, annealing temperatures (*T*_*a*_) and PCR conditions are described in Additional file 1.

#### In vitro transcription, T-cleavage assay and mass spectrometry

Unincorporated dNTPs were dephosphorylated by adding 1.7μL H_2_O and 0.3 units of shrimp alkaline phosphatase (SAP; SEQUENOM, Inc., San Diego, CA). The reaction mixture was incubated at 37°C for 20 minutes and the SAP was then heat inactivated for 10 minutes at 85°C. Typically, 2μL of the PCR were used directly as a template in a 5μL transcription reaction. Twenty units of T7 R&DNA polymerase (Epicentre, Madison, WI) were used to incorporate dTTP in the transcripts. Ribonucleotides were used at 1 mmol/L and the dNTP substrate at 2.5 mmol/L. In the same step, the *in vitro* transcription RNase A (SEQUENOM) was added to cleave the *in vitro* transcript (T-cleavage assay). The mixture was further diluted with H_2_O to a final volume of 27μL. Twenty-two nanoliters of cleavage reaction were robotically dispensed (nanodispenser) onto silicon chips preloaded with matrix (SpectroCHIP; SEQUENOM, San Diego). Mass spectra data were collected using a MassARRAY Compact MALDI-TOF (SEQUENOM) and spectra’s methylation proportions were generated by the Epityper software v1.0 (SEQUENOM, San Diego).

### Cell signaling and pathway analysis

Gene networks and canonical pathways displaying hypermethylated genes in lymph node metastasis were identified using the Pathway Studio® software version 7.1 (Mammal) database (Ariadne Genomics, Inc., Rockville, USA). The functional analysis identified the biological perspective of the genes that were most relevant to the data sets and facilitated the understanding beyond their functional link to breast neoplasm and metastasis.

### Statistical methods

Data analysis was performed using the SPSS software (Statistical Software Package for Windows, version 19). Distribution of data was analyzed by Kolmogorov-Smirnov test that demonstrated our data set was not normally distributed (*P* < 0.001). Quantitative methylation profile of the twelve candidate genes were compared among primary tissue, adjacent matched normal tissue and their matched lymph node metastasis using the two-way hierarchical cluster analysis. The CpG sites for each gene were clustered based on pair-wise Euclidean distances and linkage algorithm for all studied samples according to the previously developed method by Gene Expression Statistical System (GESS) version 7.1.19 (NCSS, Kaysville, Utah, USA) and the statistical differences between mean methylation quantity of the informative CpG sites per genes were identified using Mann-Whitney-U-Test. The non-parametric Spearman's rho test was performed to find out significance clinical-pathological parameters.

## Results

### Quantitative methylation profiles of the 12 breast cancer candidate genes promoter using MALDI-TOF MS

The methylation profiles of 12 breast cancer candidate genes in matched primary tumors, normal tissue and their matched lymph node metastasis from 24 breast cancer patients were assessed. For all the genes, one amplicon per gene and 250 CpG sites in total per sample (total of 16,250 sites in 65 analyzed samples) were analyzed (Table 2; Additional file 2.)

**Table 2:** High-throughput methylation analysis of CpG sites per amplicon for the 12 candidate genes

**Genes**	**Amplicon size (bp)**	**Total No. of CpG sites in amplicon**	**No. of analyzed CpG sites in amplicon**	**No. of analyzed CpG sites per amplicons**	
				**Single sites**	**Composite sites**
*APC*	420	26	11	9	2
*BIN1*	330	32	12	3	9
*BMP6*	397	37	26	9	17
*BRCA1*	413	30	15	10	5
*CST6*	445	49	19	11	8
*ESR-b (ER beta)*	374	30	13	6	7
*GSTP1*	381	23	16	11	5
*P14*	425	36	22	5	17
*P16 (CDKN2A)*	580	62	38	12	26
*P21 (CDKN1A)*	419	30	9	8	1
*PTEN*	451	34	29	10	19
*TIMP3v*	441	51	40	12	38

The *in silico* digestion was performed for the T-cleavage assay. The percentage of total CpG sites in the amplicon is divided into single sites (single CpG sites) and composite sites (two or more adjacent CpG sites fall within one fragment, or when fragment masses are overlapping).

The different levels of methylation between studied samples were identified using the two-way hierarchical cluster analysis. The methylation cluster analysis for each individual studied gene is illustrated in Additional file 2.

#### Methylation alterations of the primary tumor tissue

**Figure 2:**
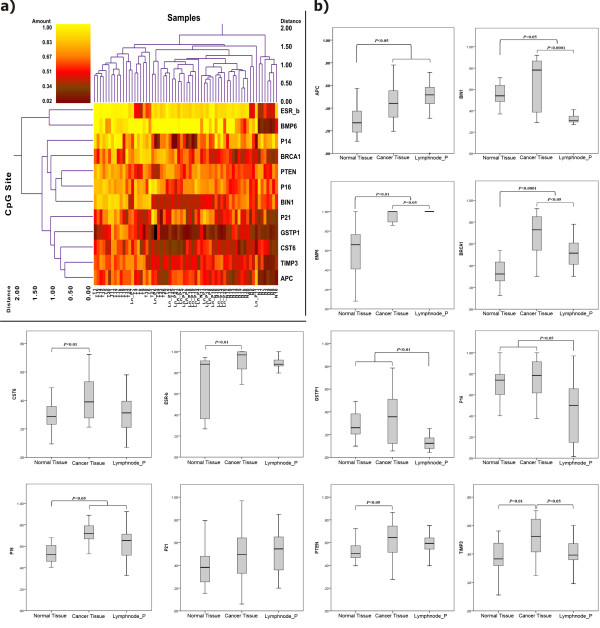
** a) Double dendrogram presents the methylation profiles of the twelve candidate genes in tumor tissue, matched normal tissue and lymph nodes metastasis (Red clusters indicate 0% methylated, yellow clusters indicate 100% methylated, color gradient between red and yellow indicates methylation ranging from 0-100).b)** Comparison between quantitative analysis (0%-100%) of methylation for the candidate genes in the studied cohort (* significant correlation; Mann-Whitney U Test).

Hierarchical cluster analysis profiling of the promoter alterations and the significance of the alterations are shown in Figure 2(a) for the 12 studied genes.

Methylation quantification of the 12 candidate genes revealed significantly higher methylation proportion for promoter regions of *APC, BIN1, BMP6, BRCA1, CST6, ESR-b, P16, PTEN* and *TIMP3* in primary tumor tissue versus matched normal tissue (*P* < 0.05, *P* < 0.05, *P* < 0.01, *P* < 0.0001, *P* < 0.01, *P* < 0.01, *P* < 0.05, *P* < 0.05, *P* < 0.01 ; respectively). Methylation analysis of *GSTP1, P14* and *P21* in primary tumor tissue showed slightly higher methylation proportion in comparison to the matched normal tissue, however, the differences were not significant (Figure 2b).

#### Methylation alterations of the matched lymph node metastasis

Comparison of methylation profiles of the 12 breast cancer candidate genes in the paired lymph node metastasis to the matched normal tissue showed significantly higher methylation levels for *APC, BMP6, BRCA1* and *P16* genes (*P* < 0.05 and *P* < 0.01, *P* < 0.0001, *P* < 0.05; respectively). The lymph node metastasis was even more hypermethylated for *BMP6* than the primary tumor tissue (*P* < 0.05). The promoter region of *BIN1*, *BMP6*, *BRCA1*, *GSTP1*, *P14* and *TIMP3* showed significantly lower methylation ratio in the lymph node metastasis than the primary tissue ( *P* < 0.0001, *P* < 0.05, *P* < 0.05, *P* < 0.01, *P* < 0.05, *P* < 0.05; respectively).

Comparing the lymph node metastasis to the matched normal tissue, the former revealed differentially lower methylation ratio for *BIN1, GSTP1* and *P14* promoter regions (*P* < 0.05, *P* < 0.01, *P* < 0.05; respectively) (Figure 2).

#### Relationship of methylation alterations within the promoter region of 12 breast cancer candidate genes and clinicopathological parameters

Clinicopathological parameters with the methylation proportion of the 12 candidate genes in primary tumor tissue and matched lymph node metastasis were analyzed. The analyses showed significant correlation between higher methylation level of *GSTP1* and increasing the histological grade in the primary tumor tissue (Spearman's rho test; *P* < 0.05). The higher methylation proportion of *BIN1* showed significant correlation with expressed ER in primary tumor tissue (Spearman's rho test; *P* < 0.01). Methylated *TIMP3* revealed significant correlation to the primary tumor tissue lacking expression of PR (Spearman's rho test; *P* < 0.05). The increase of methylation of *P21* showed an inverse correlation with the expression of C-ERB2 in the primary tumor tissue (Spearman's rho test; *P* < 0.05). We could not find any significant correlations between methylation alterations in either of the matched lymph node metastasis and clinicopathological parameters.

## Discussion

Aberrant methylation profiles and silencing of a small subset of tumor suppressors and cancer related genes involved in both the primary tumor and lymph node metastasis has been investigated for breast cancer [11,22-24]. Several studies on breast cancer revealed some similarities and differences between promoter methylation pattern of the studied genes in primary and lymph node metastatic [16,19,25]. These investigations highlight the important role of aberrant promoters’ methylation in the metastatic process. In the present study, we implemented the methylation signature of the 12 breast cancer candidate genes (*APC, BIN1, BMP6, BRCA1, CST6, ESR-b, GSTP1, P14, P16, P21, PTEN* and *TIMP3*) by comparing lymph nodes metastasis to their matched primary tumor tissues and normal tissues from the same breast cancer patients.

The quantitative methylation analysis of the 12 studied genes in the present cohort showed higher methylation proportion for the primary tumor tissue versus matched normal tissue and the differences were significant for *APC, BIN1, BMP6, BRCA1, CST6, ESR-b, P16, PTEN* and *TIMP3* promoter regions (*P* < 0.05, *P* < 0.05, *P* < 0.01, *P* < 0.0001, *P* < 0.01, *P* < 0.01, *P* < 0.05, *P* < 0.05, *P* < 0.01; respectively). Among the significant methylated genes, *APC, BMP6, BRCA1* and *P16* represented higher methylation proportions in matched lymph node metastasis compared to those of the normal tissue (*P* < 0.05 and *P* < 0.01, *P* < 0.0001, *P* < 0.05; respectively). Present findings provided evidence of differences in methylation status between primary tumors and their corresponding matched lymph nodes and demonstrated methylation heterogeneity between primary tumors and metastatic lesion which are in line with previous reports described about primary tumor and metastasis in breast, gastric and colorectal cancers [13,26,27]. The results also indicated that some of the cancer specific changes become altered over the metastasis procedure. The mechanism for losing or gaining methylation in lymph nodes metastatic is still not clear while the alteration of the methylation signature from primary to metastasis might be due to the adaptation response of the disseminated cells to the microenvironment at the site of colonization [28].

**Figure 3:**
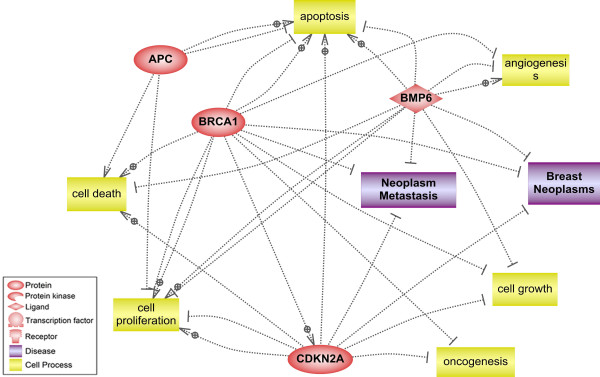
Pathway analysis of the hypermethylated genes in lymph node metastasis including their biological processes and association to breast carcinogenesis and neoplasm metastasis.

The molecular function, biological processes and contribution of the studied genes to developing metastasis were analyzed by ResNet® 7 (Mammal). The pathway analysis revealed that *BMP6BRCA1* and *P16* have a role in prevention of neoplasm metastasis (Figure 3). The relation of DNA methylation for *BRCA1* and *P16* with tumor recurrence has been reported with a high value in breast cancer patients [29]. Moreover, association of *P16* hypermethylation with cancer progression and lymph node invasion has been shown in different studies [30,31]. Then, the aberrant methylation signatures of these genes found in the metastatic lymph node can provide a further clue to establish useful biomarkers for screening metastasis in breast cancer. Such biomarkers will need to be additionally explored for the potential opposing functional effects of specific hypermethylated CpG sites on transcriptional activity as well as for absolute percent methylation cutoffs for each breast tumor type that would enable reliable utility of them in the clinical laboratory setting. The possibility of using these hypermethylated genes as biomarkers in our previous study was investigated with the aim of developing a blood based panel for plasma and serum samples of breast cancer patients [18]. Thus the detection of these three hypermethylated genes either as tissue specific biomarkers or as circulating biomarkers may give insight into the prognosis and therapeutic management of the breast cancer patients. Moreover, we identified significantly greater hypermethylation proportion of *BMP6* in the lymph node metastasis than their primary tumor tissue (*P* < 0.05). *BMP6* is a member of TGF-β super family and critically involved in many developmental processes [32]. Recently, the close association of *BMP6* with progression of tumorigenesis and regulation of invasion for tumor cells has been reported [33]. It has been assumed that demethylation of *BMP6* and re-expression of this gene might modulate metastasis and invasion in breast cancer [34,35].

## Conclusions

In conclusion, the present study showed aberrant tumor-specific methylation alterations for *APC, BIN1, BMP6, BRCA1, CST6, ESR-b, GSTP1, P14, P16, P21, PTEN* and *TIMP3* in the studied cohort. Additionally, we identified methylation heterogeneity between primary tumors and metastatic lesions. The contribution of aberrant methylation alterations of *BMP6*, *BRCA1* and *P16* genes in lymph node metastasis might provide a further clue to establish useful biomarkers for screening metastasis, which might improve prognosis and therapeutic management of the breast cancer patients.

## Competing interest

There is no conflict of interest in the present study.

## Authors’ contributions

XYZ and ZB conceived and designed the experiment, ZB carried out experiments, ZB and RR analyzed the data, HZ and QL, provided samples, JB, PL, PT, XYZ material and analysis tools. ZB and XYZ were involved in writing the paper; all authors had final approval of the submitted and published versions.

## Pre-publication history

The pre-publication history for this paper can be accessed here:

http://www.biomedcentral.com/1471-2407/12/244/prepub

## Supplementary Material

Additional file 1 **Supplementary Data 1.** The sequence of PCR primers, PCR conditions and complete data for high-throughput methylation analysis of informative CpG sites in 12 breast cancer-related genes, including: gene location, amplicon size and two-way hierarchical cluster analysis are illustrated in dataset. Click here for file

Additional file 2 **Supplementary Data 2.** High-throughput methylation analysis of CpG sites for 12 candidate genes that are related to breast cancer. Click here for file
