# Supplementary material for: Methylation signature of lymph node metastases in breast cancer patients
Source: BMC Cancer. 2012 Jun 13;12:244. doi: 10.1186/1471-2407-12-244 (PMC3437205; doi:10.1186/1471-2407-12-244)
Supplement: Additional file 1 — Supplementary Data 1. The sequence of PCR primers, PCR conditions and complete data for high-throughput methylation analysis of informative CpG sites in 12 breast cancer-related genes, including: gene location, amplicon size and two-way hierarchical cluster analysis are illustrated in dataset. [file 1471-2407-12-244-S1.doc]

**Supplementary Data 1**

The sequence of PCR tagged primers for *in vitro* transcription and PCR conditions

For the PCR on bisulfite-treated genomic DNA (gDNA), the following PCR conditions were used: 1x: 95°C for 10 min; 48x: 95°C for 30s, Ta for 40s, 72°C for 1 min; 1x 72°C for 5 min. The PCR cocktail was: 2μL DNA (2.00μL of at least 10 ng/μL DNA for a final concentration of 2ng/μL per reaction) in a 10μL total volume using 1pmol of each primer, 200μM dNTP, 0.2 unit Hot Start Taq DNA polymerase, 1.5mM MgCl_2_ and the buffer supplied with the enzyme.

For re-amplification, 2μL of first round PCR reaction (25–250ng amplicon) was directly used as template with the same primer pairs under the same PCR cycling conditions.

| ***Gene*** | **Primer** | **Sequence (5’→3’)** | **Length** | **T_a_** | **Prodoct Size (bp)** |
| --- | --- | --- | --- | --- | --- |
| *APC* | tag-EN1-FW | AGGAAGAGAGATTGTTTTTTTGTGTTGTAAAAATTAT | 27+10 | 58 | 420 |
|  | T7-EN1-RV | CAG**TAATACGACTCACTATAGGGAGA**AGGCTCACCTCCATTCTATCTCCAATAAC | 24+31 |  |  |
| *BIN1* | tag-EN1-FW | AGGAAGAGAGGGAGGTGAGTTTTTGGAA | 18+10 | 58 | 330 |
|  | T7-EN1-RV | CAG**TAATACGACTCACTATAGGGAGA**AGGCTCTACCTTTTAAAAAACCACTCC | 22+31 |  |  |
| *BMP6* | tag-EN1-FW | AGGAAGAGAGGGGGTAAATTTTATGGTGGTTT | 22+10 | 57 | 397 |
|  | T7-EN1-RV | CAG**TAATACGACTCACTATAGGGAGA**AGGCTCCTTCCTAACCCTCAATCCTTA | 22+31 |  |  |
| *BRCA1* | tag-EN1-FW | AGGAAGAGAGAATTGGAGATTTTTATTAGG | 20+10 | 56 | 413 |
|  | T7-EN1-RV | CAG**TAATACGACTCACTATAGGGAGA**AGGCTAAATCTCAACRAACTCAC | 18+31 |  |  |
| *CST6* | tag-EN1-FW | AGGAAGAGAGGTTGGTAGTTTATTTTGGATAGTTT | 25+10 | 59 | 445 |
|  | T7-EN1-RV | CAG**TAATACGACTCACTATAGGGAGA**AGGCTCAAATCCCRAAATTCTCC | 18+31 |  |  |
| *ESR-b (ER beta)* | tag-EN1-FW | AGGAAGAGAGTTTAGTTGTTGGTTTTTTGGAT | 22+10 | 58 | 374 |
|  | T7-EN1-RV | CAG**TAATACGACTCACTATAGGGAGA**AGGCTAAAATTTCAAACAAAATAAAACAATT | 26+31 |  |  |
| *GSTP1* | tag-EN1-FW | AGGAAGAGAGTTYGGGAGGTTGAAGTAGA | 19+10 | 60 | 381 |
|  | T7-EN1-RV | CAG**TAATACGACTCACTATAGGGAGA**AGGCTAAACAAACAACAAAAAAAAAACC | 23+31 |  |  |
| *P14^ARF^* | tag-EN1-FW | AGGAAGAGAGGTTTTTGGTAGGGTYGTGTT | 20+10 | 58 | 425 |
|  | T7-EN1-RV | CAG**TAATACGACTCACTATAGGGAGA**AGGCTACTCCTACCCCCTTAACTACAAA | 23+31 |  |  |
| *P16 (CDKN2A)* | tag-EN1-FW | AGGAAGAGAGGGTTGTTTTTGGTAGGG | 17+10 | 58 | 580 |
|  | T7-EN1-RV | CAG**TAATACGACTCACTATAGGGAGA**AGGCTATATAAACCACRAAAACCC | 19+31 |  |  |
| *P21 (CDKN1A)* | tag-EN1-FW | AGGAAGAGAGGGTAAATTTTTGTTTGTTAGAGTGG | 25+10 | 60 | 419 |
|  | T7-EN1-RV | CAG**TAATACGACTCACTATAGGGAGA**AGGCTTAACTTCRACAACTACTCACACCT | 24+31 |  |  |
| *PTEN* | tag-EN1-FW | AGGAAGAGAG GAGTGGGAATTTGGAAAGTTTTTTA | 25+10 | 58 | 451 |
|  | T7-EN1-RV | CAG**TAATACGACTCACTATAGGGAGA**AGGCTCAAAAACCCAAAAAACACCTATCT | 24+31 |  |  |
| *TIMP3* | tag-EN1-FW | AGGAAGAGAGTTTTGTTATTGGTTTGAGGG | 20+10 | 59 | 441 |
|  | T7-EN1-RV | CAG**TAATACGACTCACTATAGGGAGA**AGGCTCCAAACTCCAACTACCCA | 18+31 |  |  |


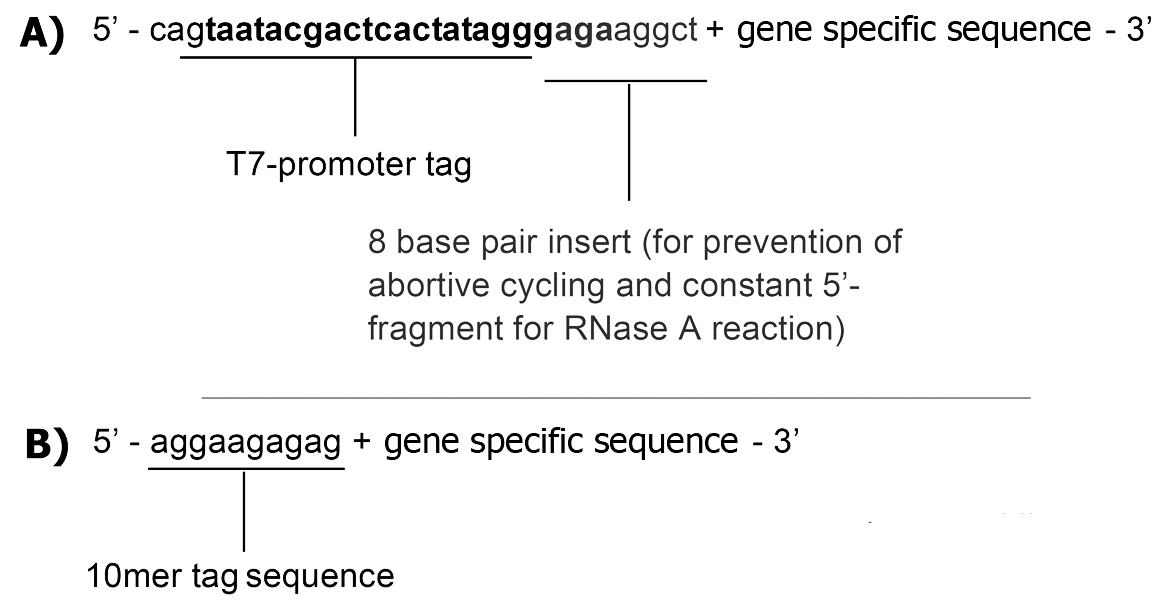


Primers for *in vitro* transcription. (A) Reverse primer with T7-promoter tag. (B) Forward primer with 10mer tag sequence as balance.
