# Supplementary material for: Methylation signature of lymph node metastases in breast cancer patients
Source: BMC Cancer. 2012 Jun 13;12:244. doi: 10.1186/1471-2407-12-244 (PMC3437205; doi:10.1186/1471-2407-12-244)
Supplement: Additional file 2 — Supplementary Data 2. High-throughput methylation analysis of CpG sites for 12 candidate genes that are related to breast cancer. [file 1471-2407-12-244-S2.doc]

**Supplementary Data**

High-throughput methylation analysis of CpG sites in 12 candidate genes related to breast cancer.

***APC* Gene**

| **Gene ID** | **Alternate gene name** | **locus** | **Function** | **Methylation effect on breast cancer** |
| --- | --- | --- | --- | --- |
| 324 | adenomatous polyposis coli | 5q21-q22 | Cell adhesion, signal transduction, stabilization of the cytoskeleton, regulation of cell cycle and apoptosis | direct |


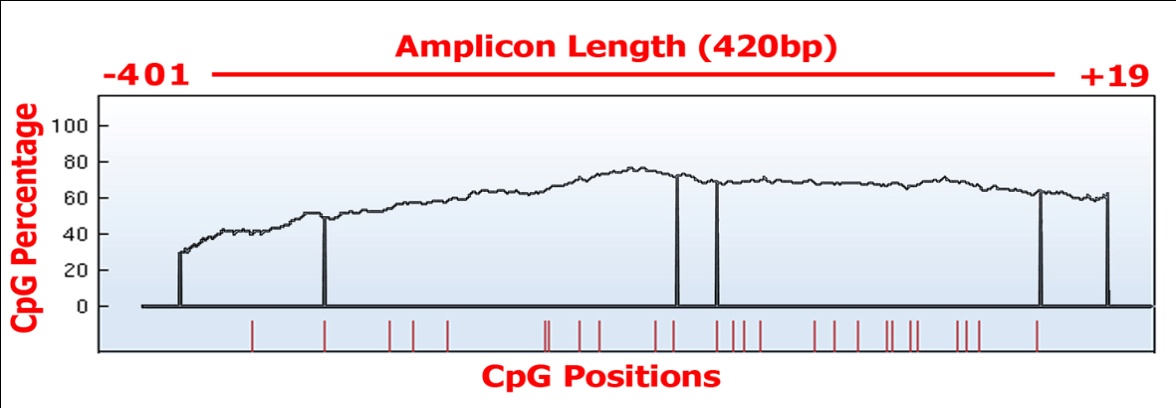


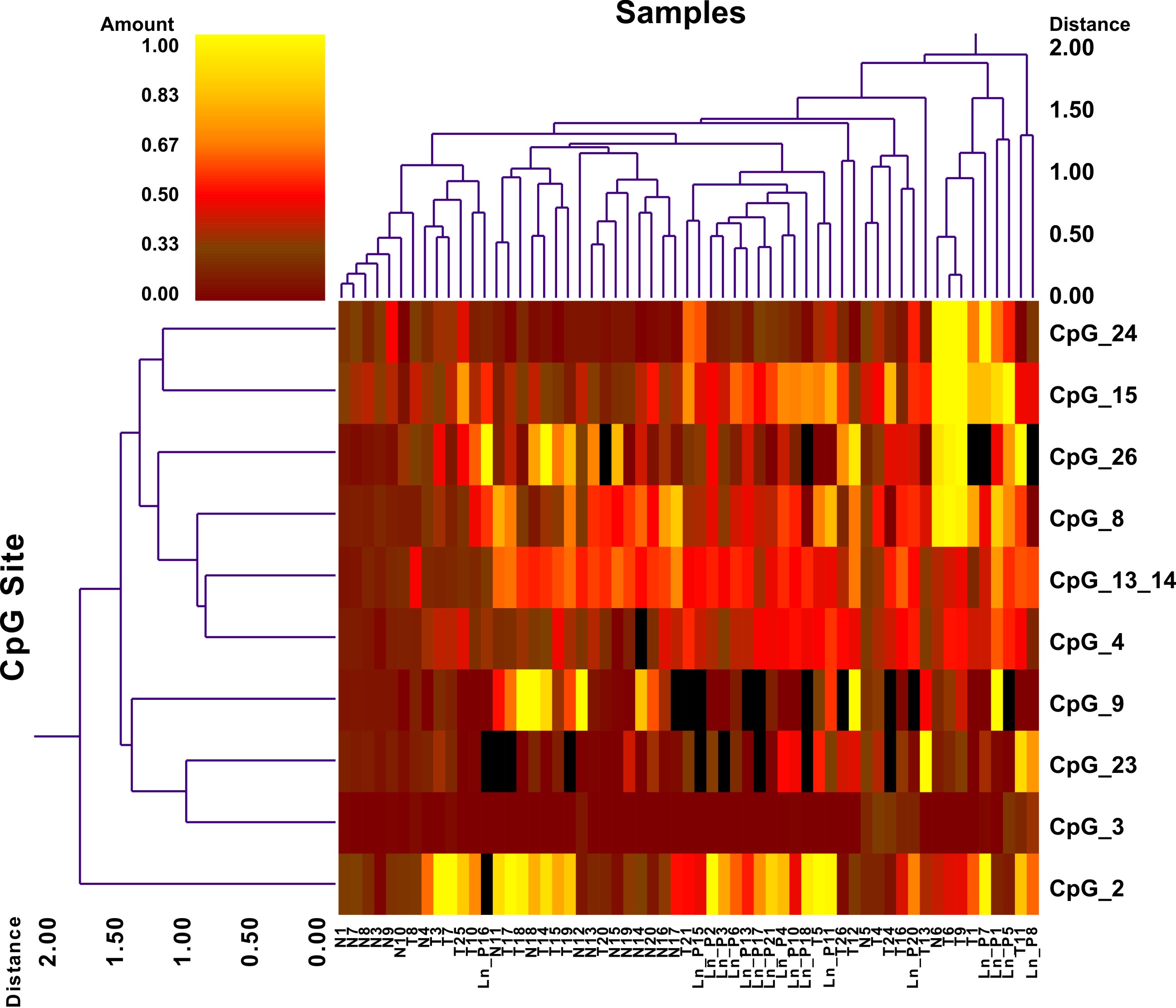


**Double dendrogram of *APC* gene:** Two-way hierarchical cluster analysis of breast cancer studied cohort. (Red clusters indicate 0% methylated, yellow clusters indicate 100% methylated, color gradient between red and yellow indicates methylation ranging from 0-100, and black clusters indicate not analyzed CpG sites).

***BIN1* Gene**

| **Gene ID** | **Alternate gene name** | **locus** | **Function** | **Methylation effect on breast cancer** |
| --- | --- | --- | --- | --- |
| 274 | Bridging integrator 1 | 2q14 | Encodes several isoforms of a nucleocytoplasmic adaptor protein, one of which was initially identified as a MYC-interacting protein with features of a tumour suppressor. | direct |


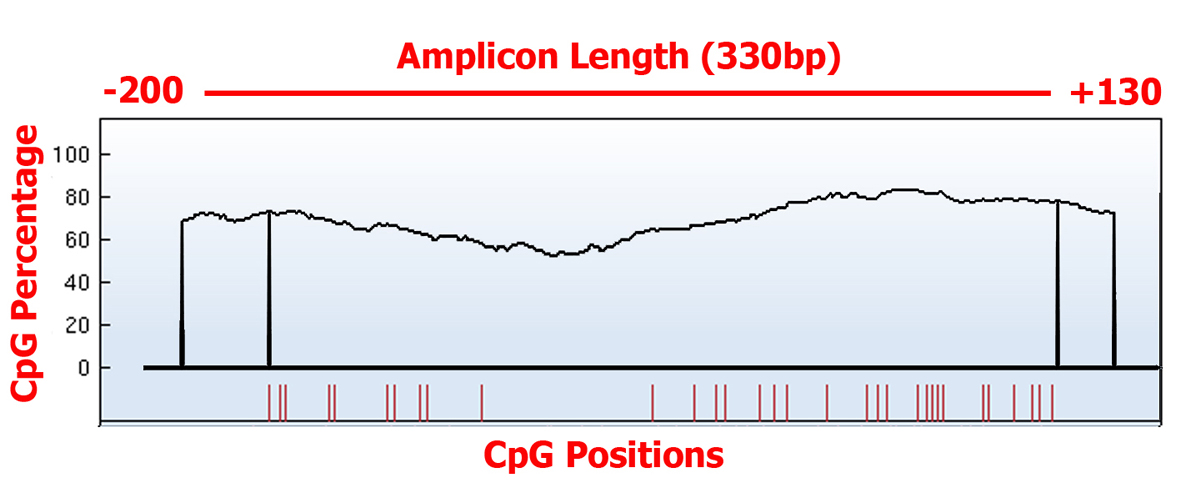


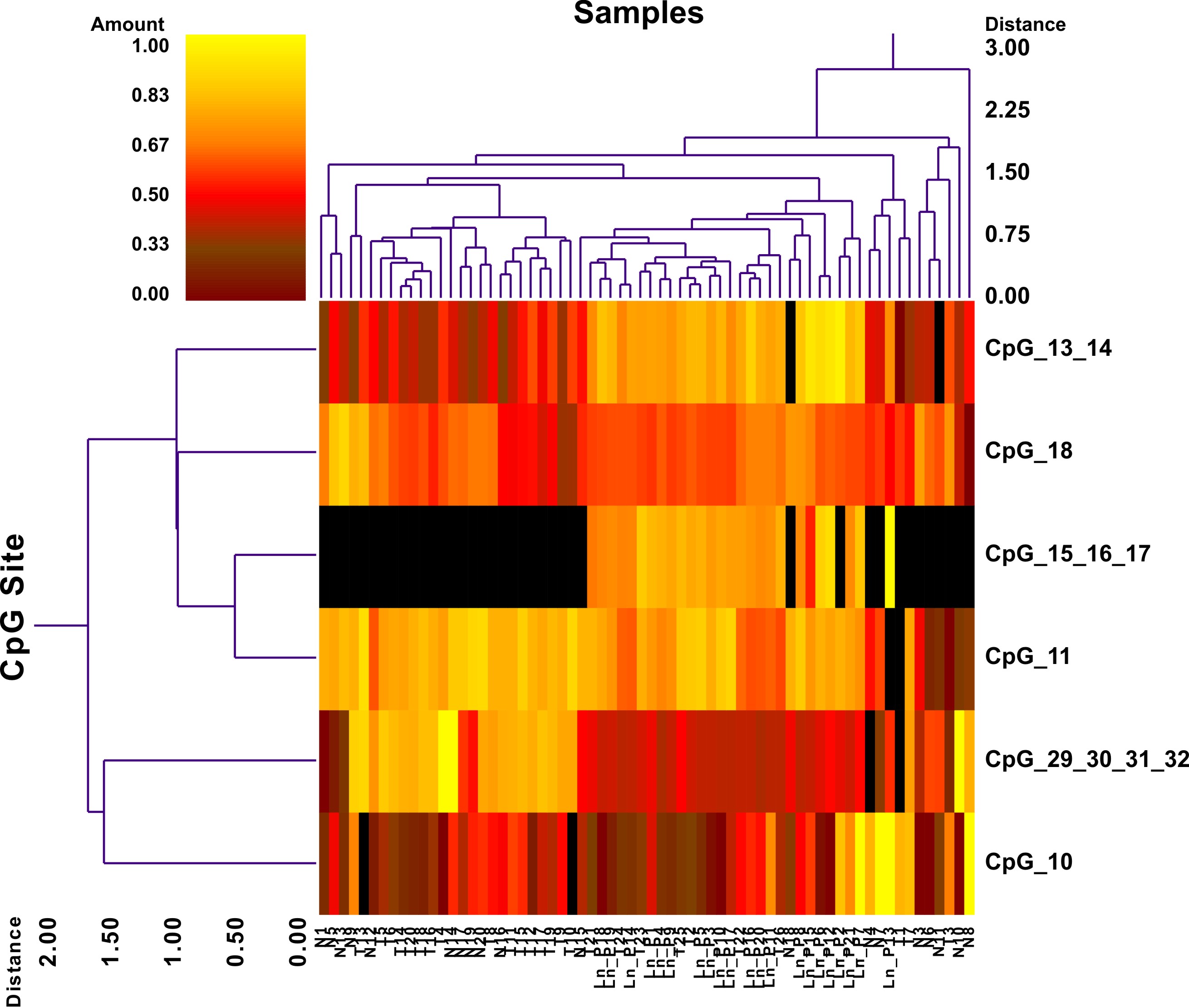


**Double dendrogram of *BIN1* gene:** Two-way hierarchical cluster analysis of breast cancer studied cohort. (Red clusters indicate 0% methylated, yellow clusters indicate 100% methylated, color gradient between red and yellow indicates methylation ranging from 0-100, and black clusters indicate not analyzed CpG sites).

***BMP6* Gene**

| **Gene ID** | **Alternate gene name** | **locus** | **Function** | **Methylation effect on breast cancer** |
| --- | --- | --- | --- | --- |
| 654 | bone morphogenetic protein 6 | 6p24-p23 | The bone morphogenetic proteins (BMPs) are a family of secreted signalling molecules. | direct |

***
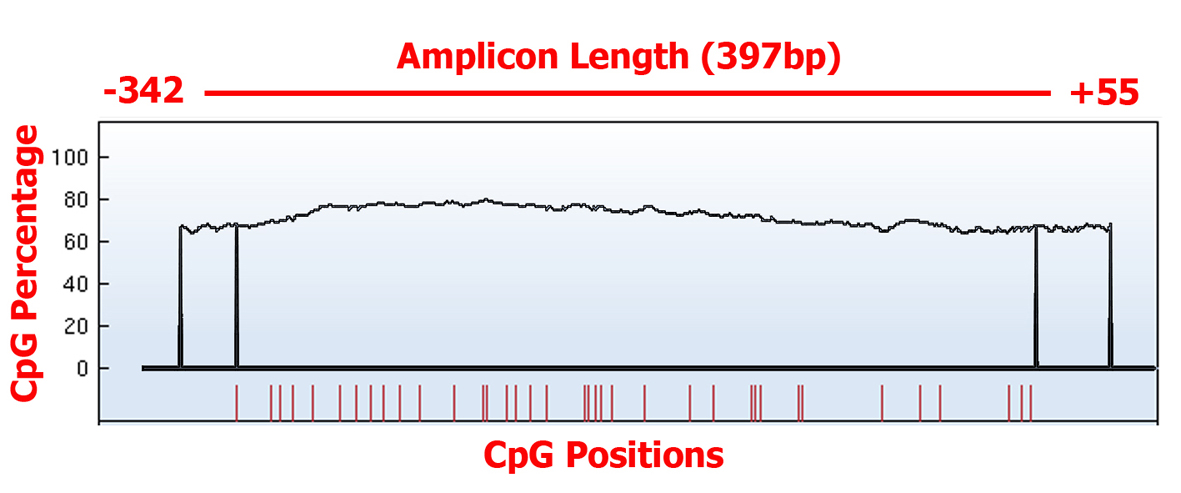
***


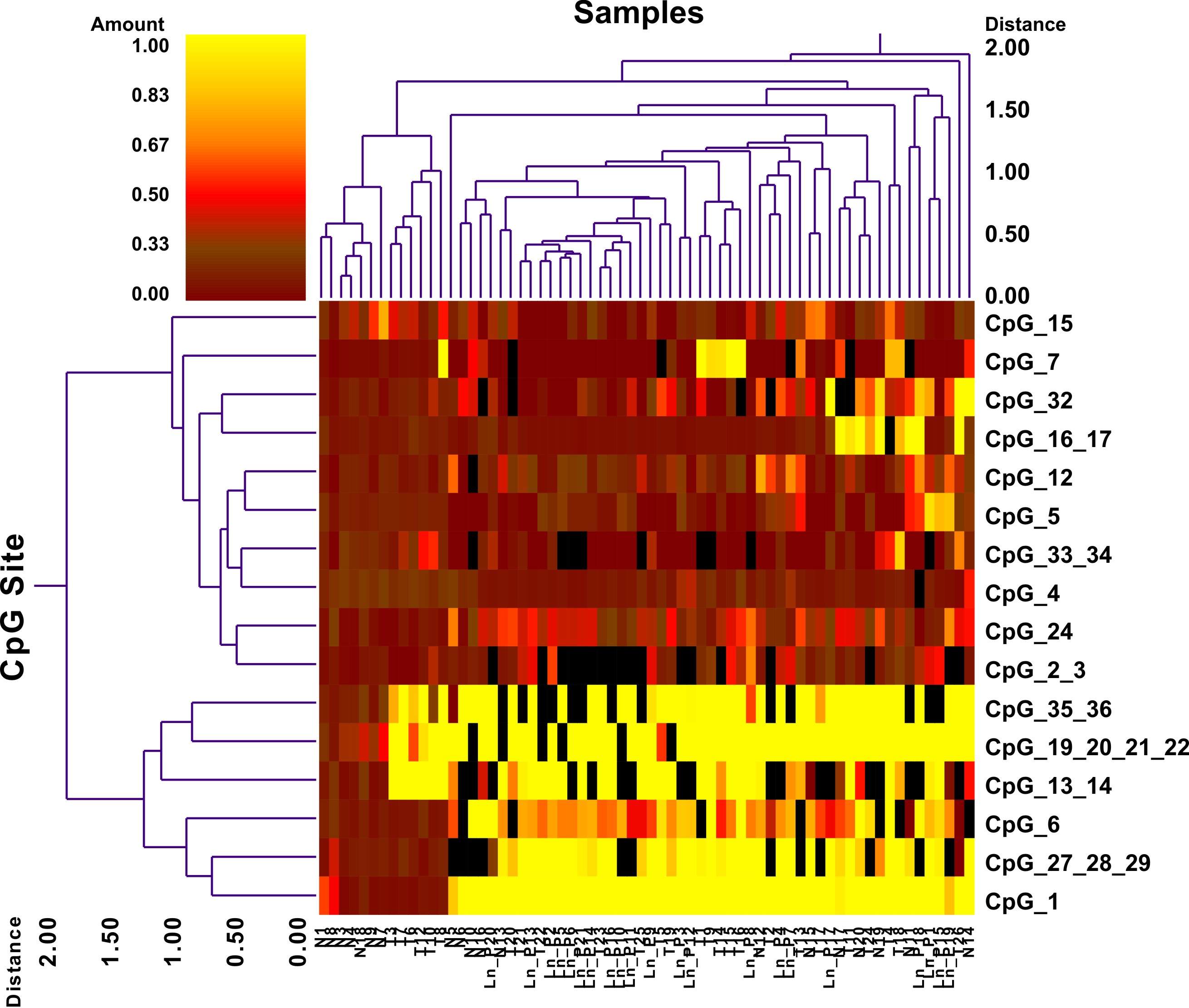


**Double dendrogram of *BMP6* gene:** Two-way hierarchical cluster analysis of breast cancer studied cohort. (Red clusters indicate 0% methylated, yellow clusters indicate 100% methylated, color gradient between red and yellow indicates methylation ranging from 0-100, and black clusters indicate not analyzed CpG sites).

***BRCA1* Gene**

| **Gene ID** | **Alternate gene name** | **locus** | **Function** | **Methylation effect on breast cancer** |
| --- | --- | --- | --- | --- |
| 672 | Breast Cancer type 1 | 17q21 | Involved in DNA repair, recombination, checkpoint control of the cell cycle and transcription. Interacts with p53, STAT-factors, SRBC, etc. | direct |

***
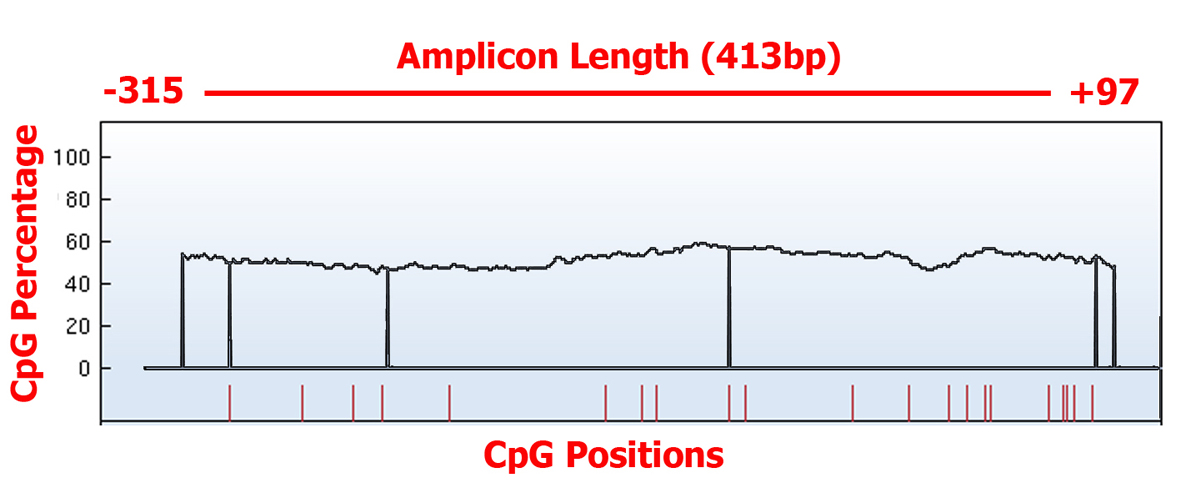
***

***
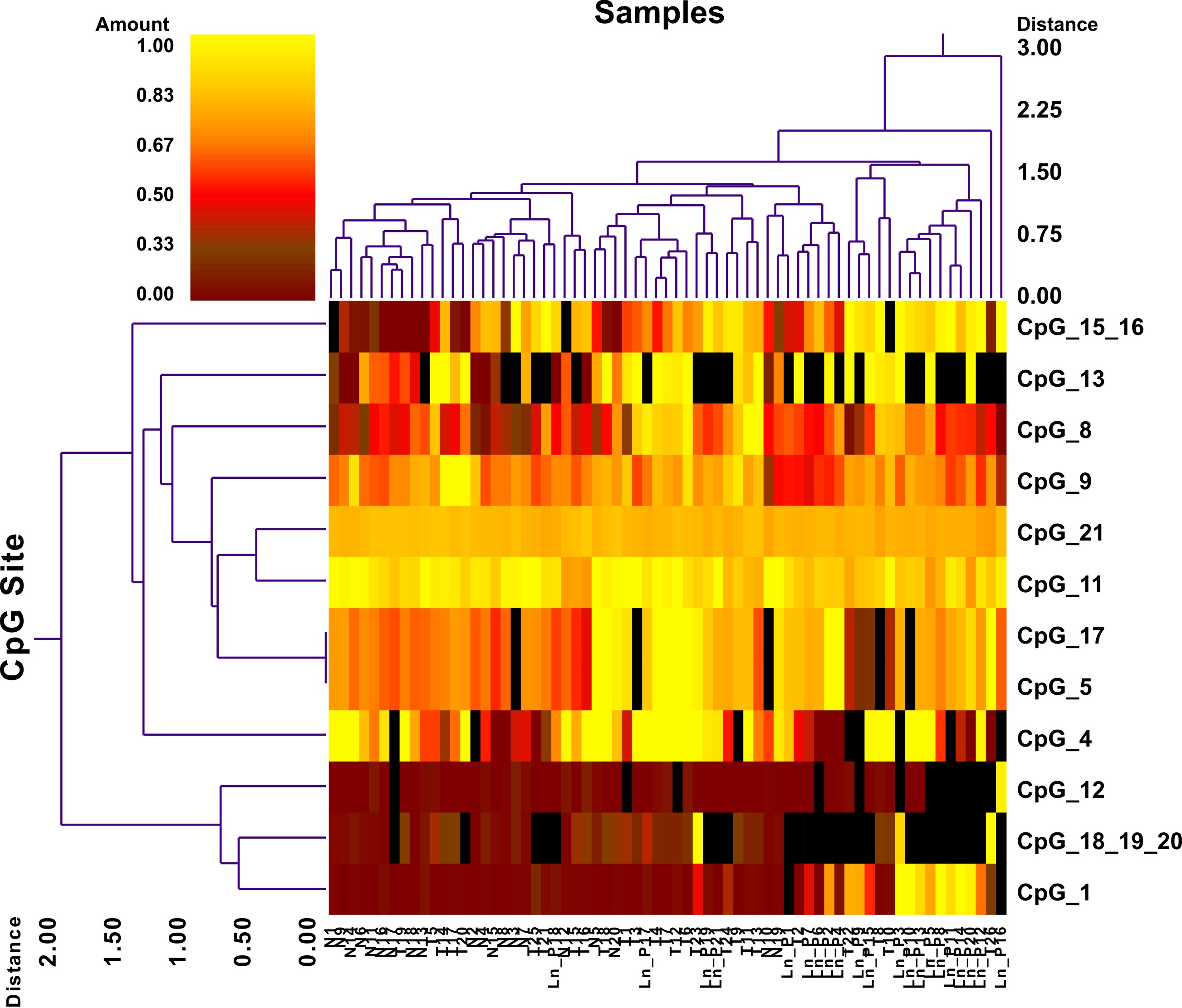
***

**Double dendrogram of *BRCA1* gene:** Two-way hierarchical cluster analysis of breast cancer studied cohort. (Red clusters indicate 0% methylated, yellow clusters indicate 100% methylated, color gradient between red and yellow indicates methylation ranging from 0-100, and black clusters indicate not analyzed CpG sites).

***CST6* Gene**

| **Gene ID** | **Alternate gene name** | **locus** | **Function** | **Methylation effect on breast cancer** |
| --- | --- | --- | --- | --- |
| 1474 | Cystatin E/M | 11q13 | This gene encodes a cystatin from type 2 family, which is down-regulated in metastatic breast tumour cells as compared to primary tumour cells. Loss of expression is likely associated with the progression of a primary tumour to a metastatic phenotype. | direct |


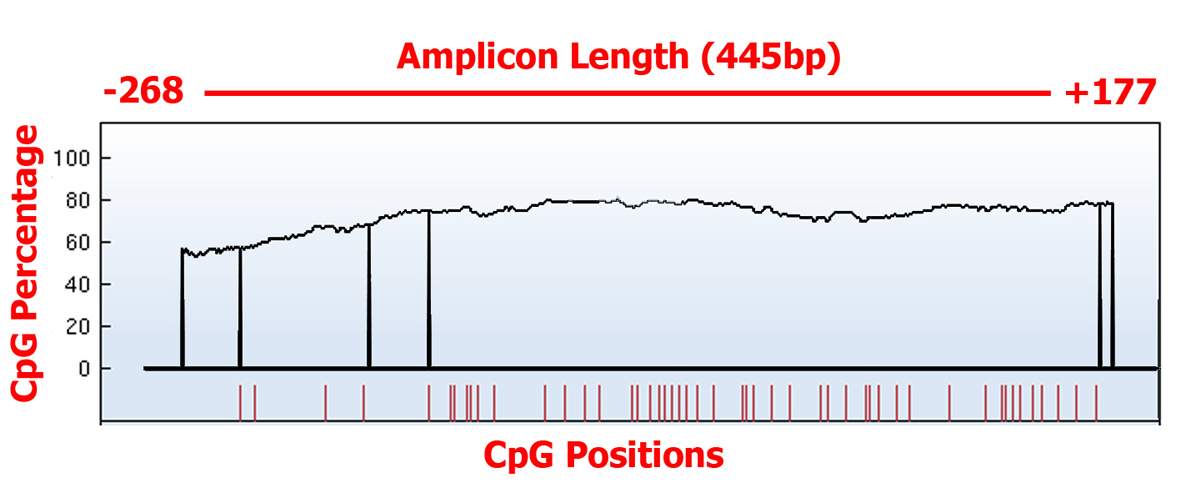


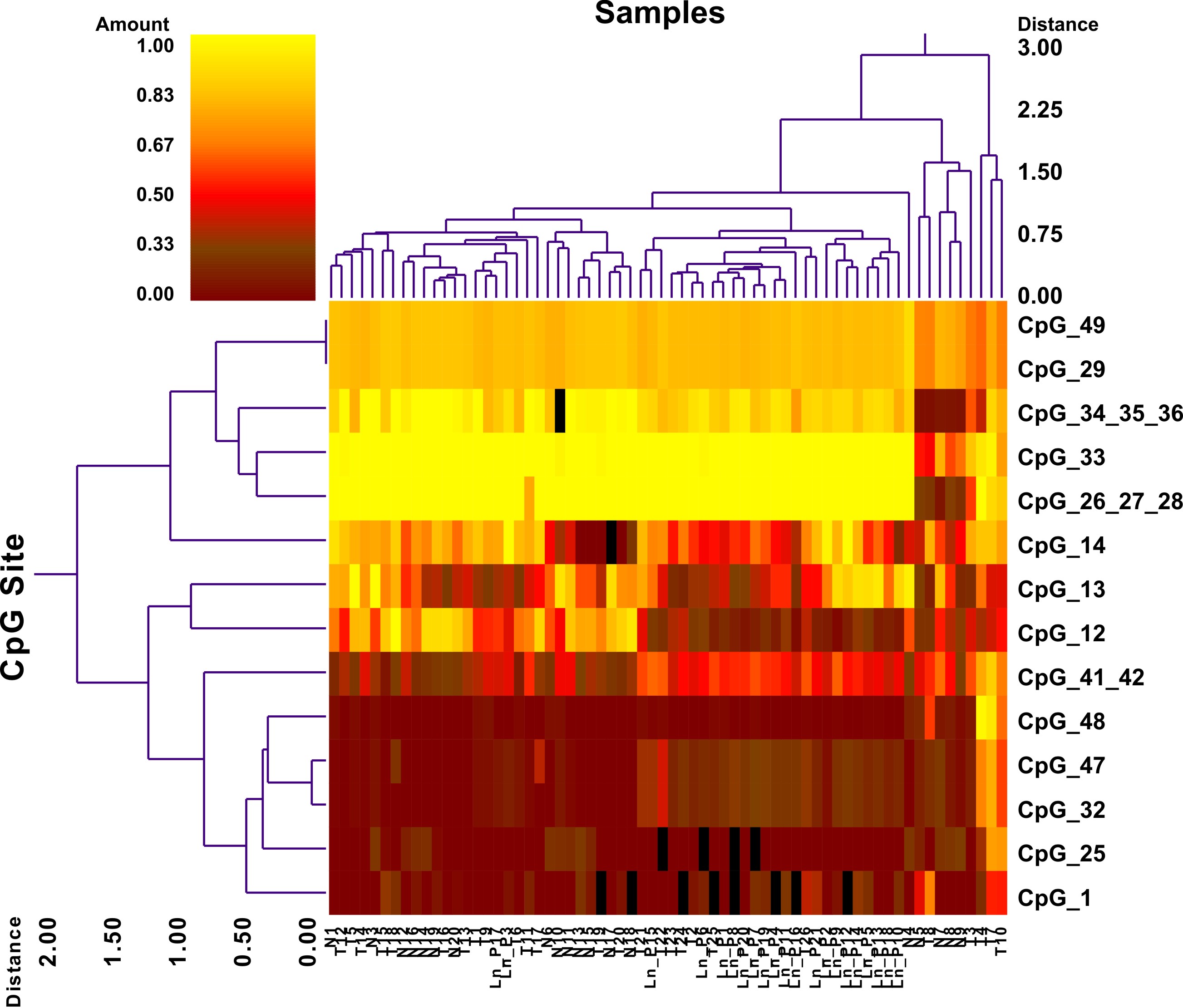


**Double dendrogram of *CST6* gene:** Two-way hierarchical cluster analysis of breast cancer studied cohort. (Red clusters indicate 0% methylated, yellow clusters indicate 100% methylated, color gradient between red and yellow indicates methylation ranging from 0-100, and black clusters indicate not analyzed CpG sites).

***ESR-b* Gene**

| **Gene ID** | **Alternate gene name** | **locus** | **Function** | **Methylation effect on breast cancer** |
| --- | --- | --- | --- | --- |
| 2100 | estrogen receptor 2 (ER beta) | 14q23.2 | Regulation of cell proliferation, predictor of endocrine therapy | direct |


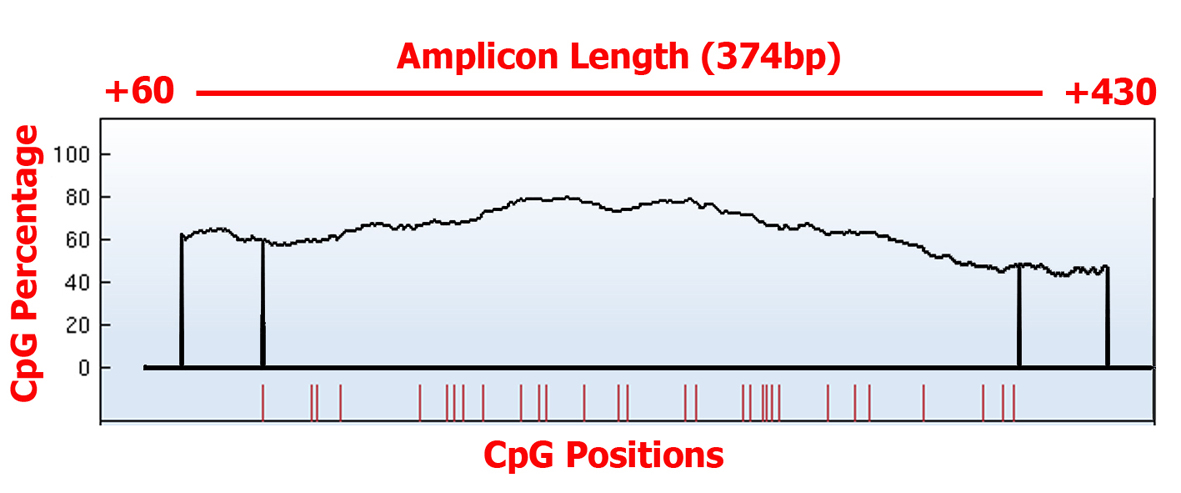


***
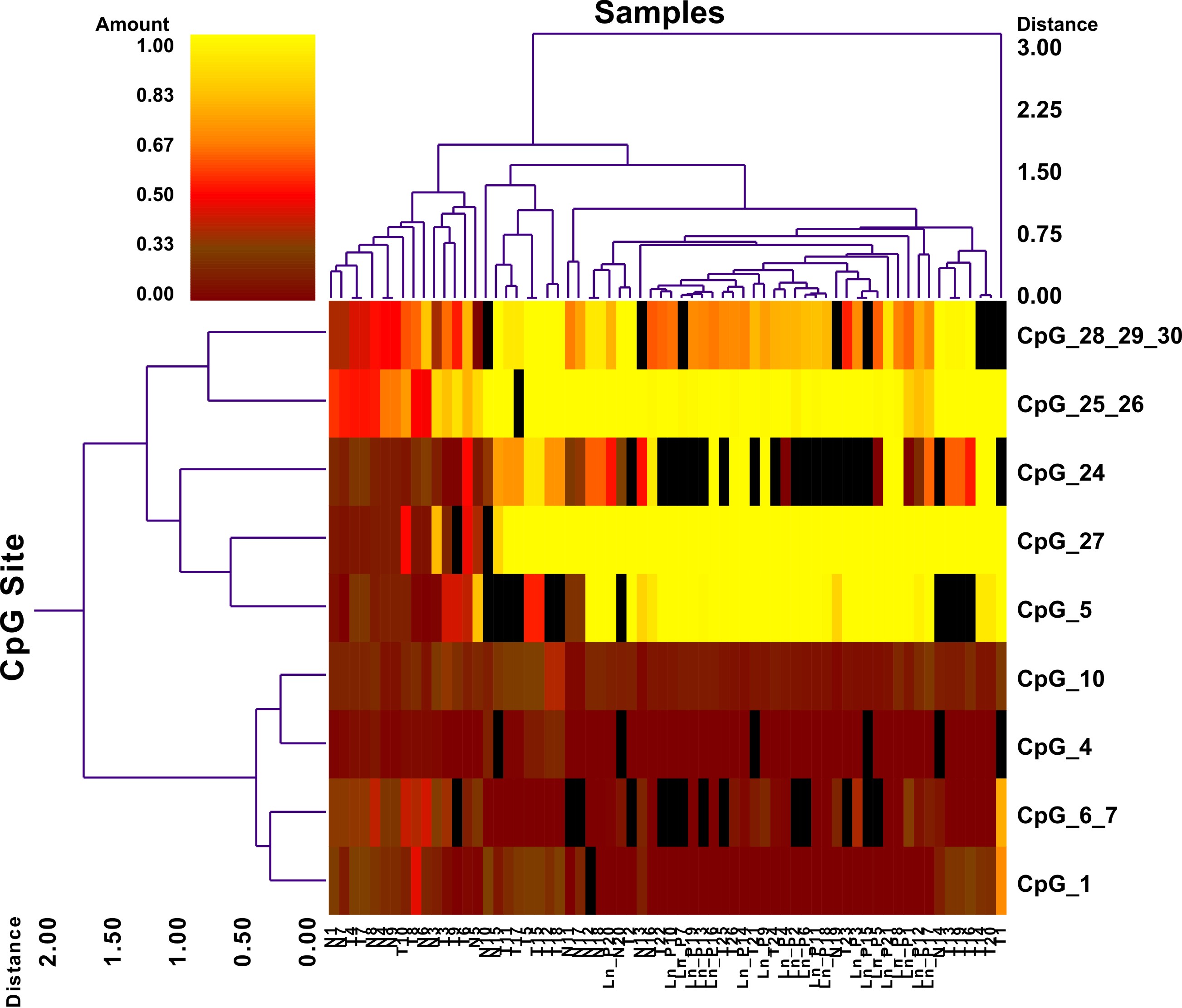
***

**Double dendrogram of *ESR-b* gene:** Two-way hierarchical cluster analysis of breast cancer studied cohort. (Red clusters indicate 0% methylated, yellow clusters indicate 100% methylated, color gradient between red and yellow indicates methylation ranging from 0-100, and black clusters indicate not analyzed CpG sites).

***GSTP1* Gene**

| **Gene ID** | **Alternate gene name** | **locus** | **Function** | **Methylation effect on breast cancer** |
| --- | --- | --- | --- | --- |
| 2950 | Glutathione S-transferase P1 | 11q13 | Carcinogen detoxification. GSTP1 is a polymorphic gene encoding active, functionally different GSTP1 variant proteins that are thought to function in xenobiotic metabolism and play a role in susceptibility to cancer, and other diseases. | direct |

***
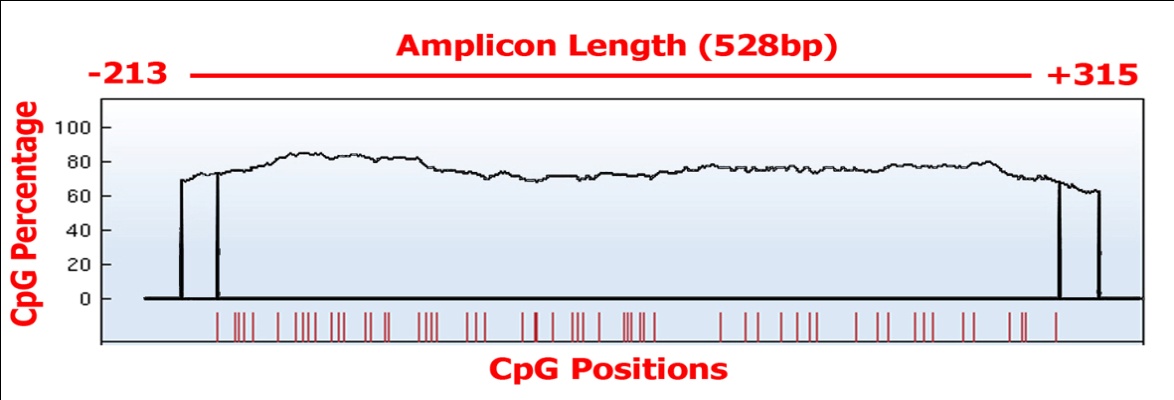
***

***
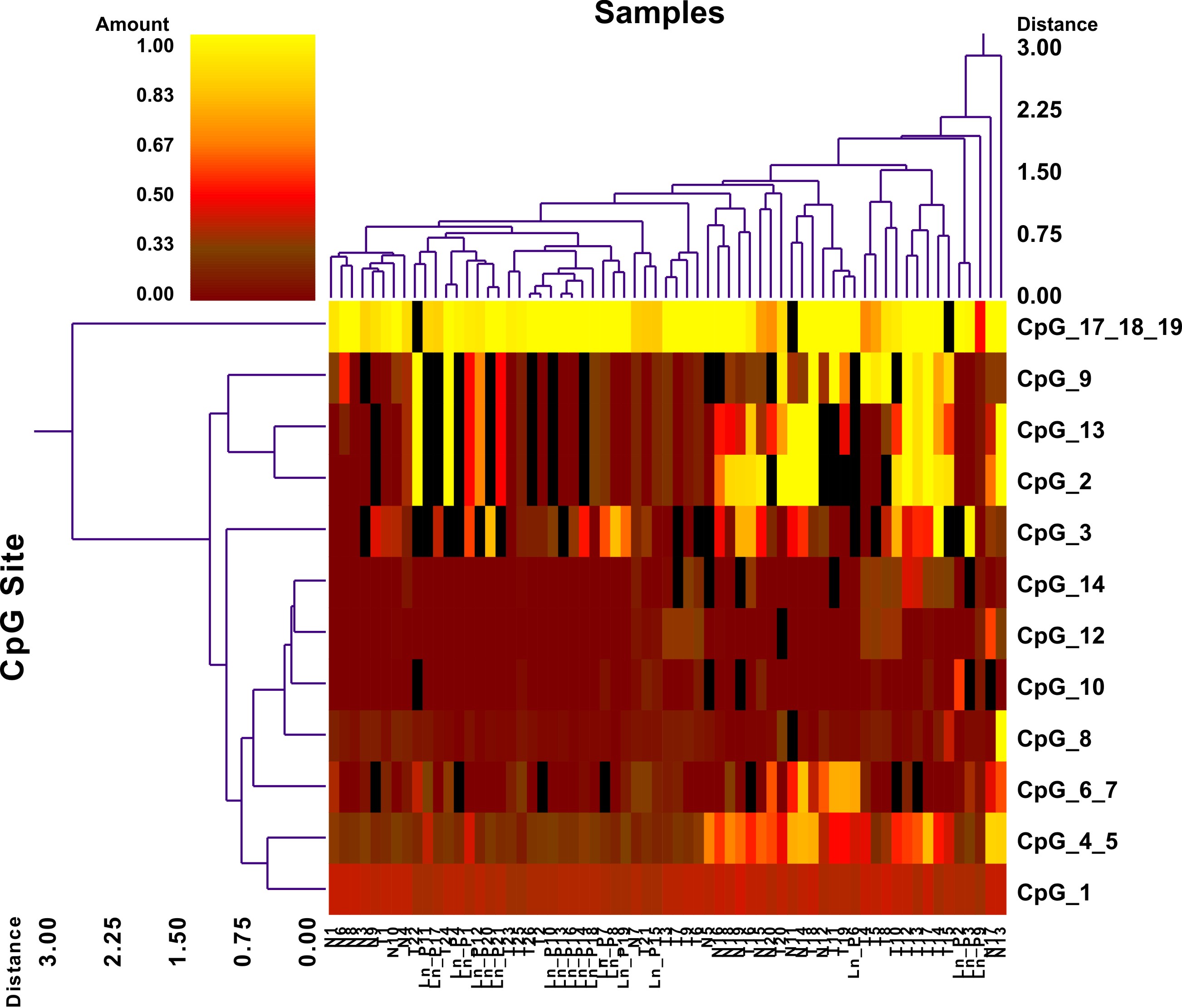
***

**Double dendrogram of *GSTP1* gene:** Two-way hierarchical cluster analysis of breast cancer studied cohort. (Red clusters indicate 0% methylated, yellow clusters indicate 100% methylated, color gradient between red and yellow indicates methylation ranging from 0-100, and black clusters indicate not analyzed CpG sites).

***P14^ARF^* Gene**

| **Gene ID** | **locus** | **Function** | **Methylation effect on breast cancer** |
| --- | --- | --- | --- |
| 1029 | 9p21 | Cell cycle regulation, involved in senescenc: This ARF product functions as a stabilizer of the tumor suppressor protein p53 as it can interact with, and sequester, MDM1, a protein responsible for the degradation of p53. | direct |


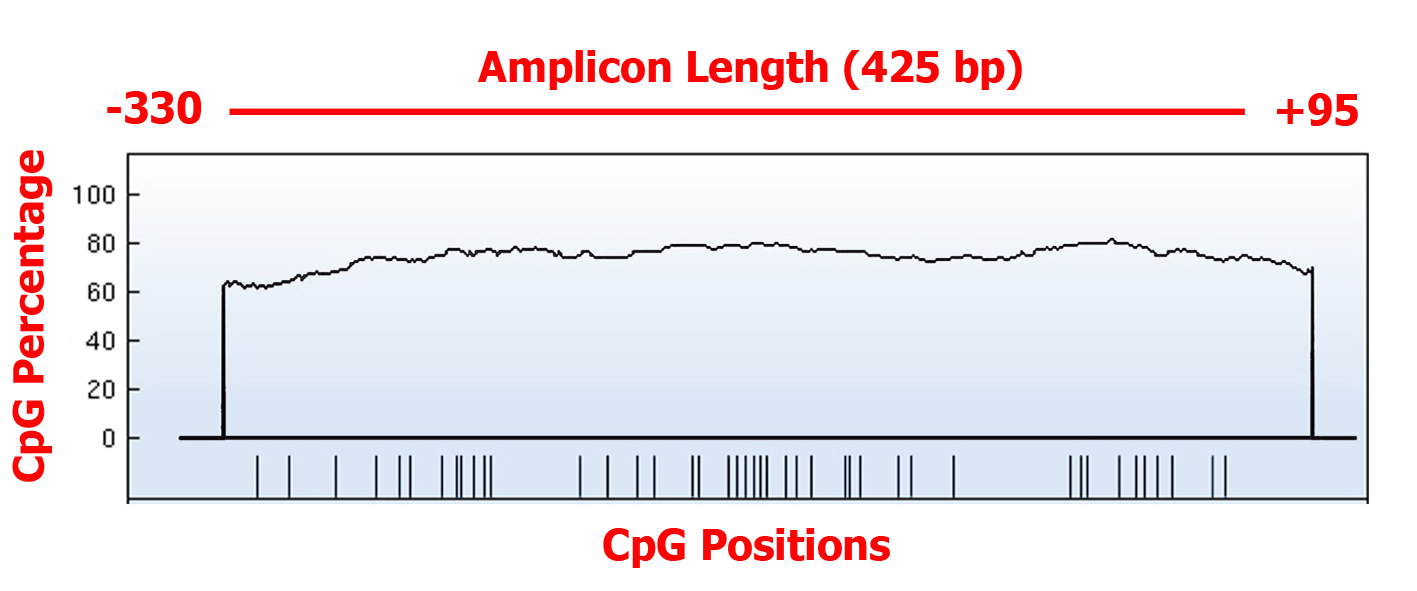


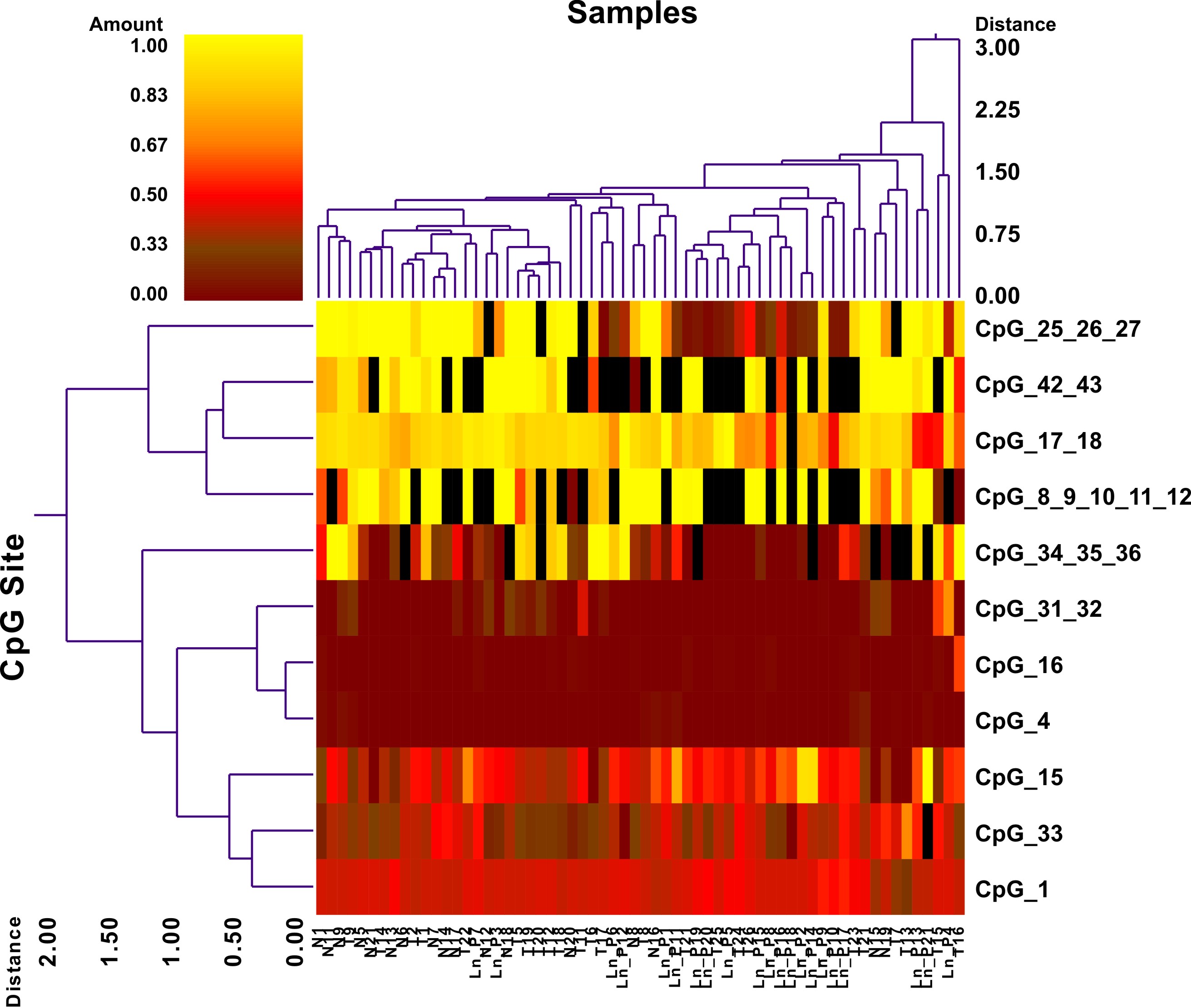


**Double dendrogram of *P14* gene:**  Two-way hierarchical cluster analysis of breast cancer studied cohort. (Red clusters indicate 0% methylated, yellow clusters indicate 100% methylated, color gradient between red and yellow indicates methylation ranging from 0-100, and black clusters indicate not analyzed CpG sites).

***P16 (CDKN2A)* Gene**

| **Gene ID** | **Alternate gene name** | **locus** | **Function** | **Methylation effect on breast cancer** |
| --- | --- | --- | --- | --- |
| 1029 | Cyclin-dependent kinase inhibitor 2A (melanoma, p16, inhibits CDK4) | 9p21 | Cell cycle regulation, involved in senescence. | direct |


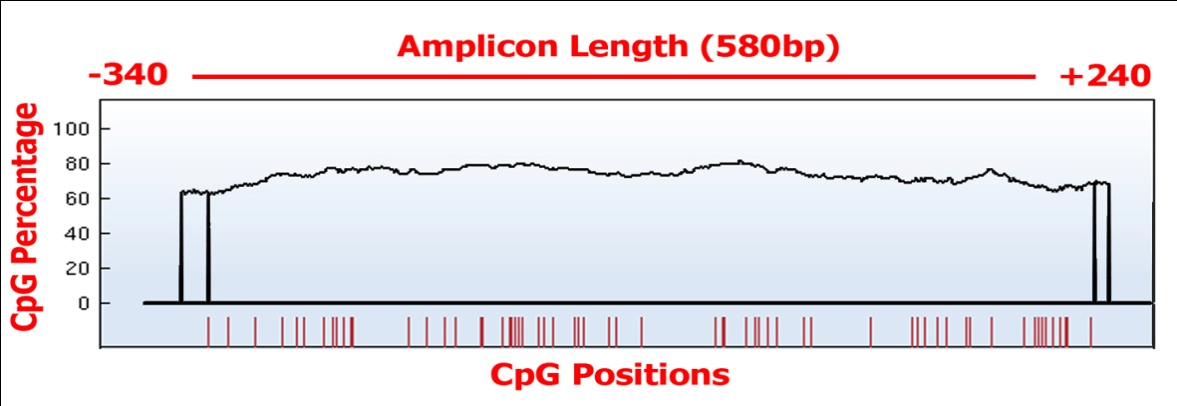


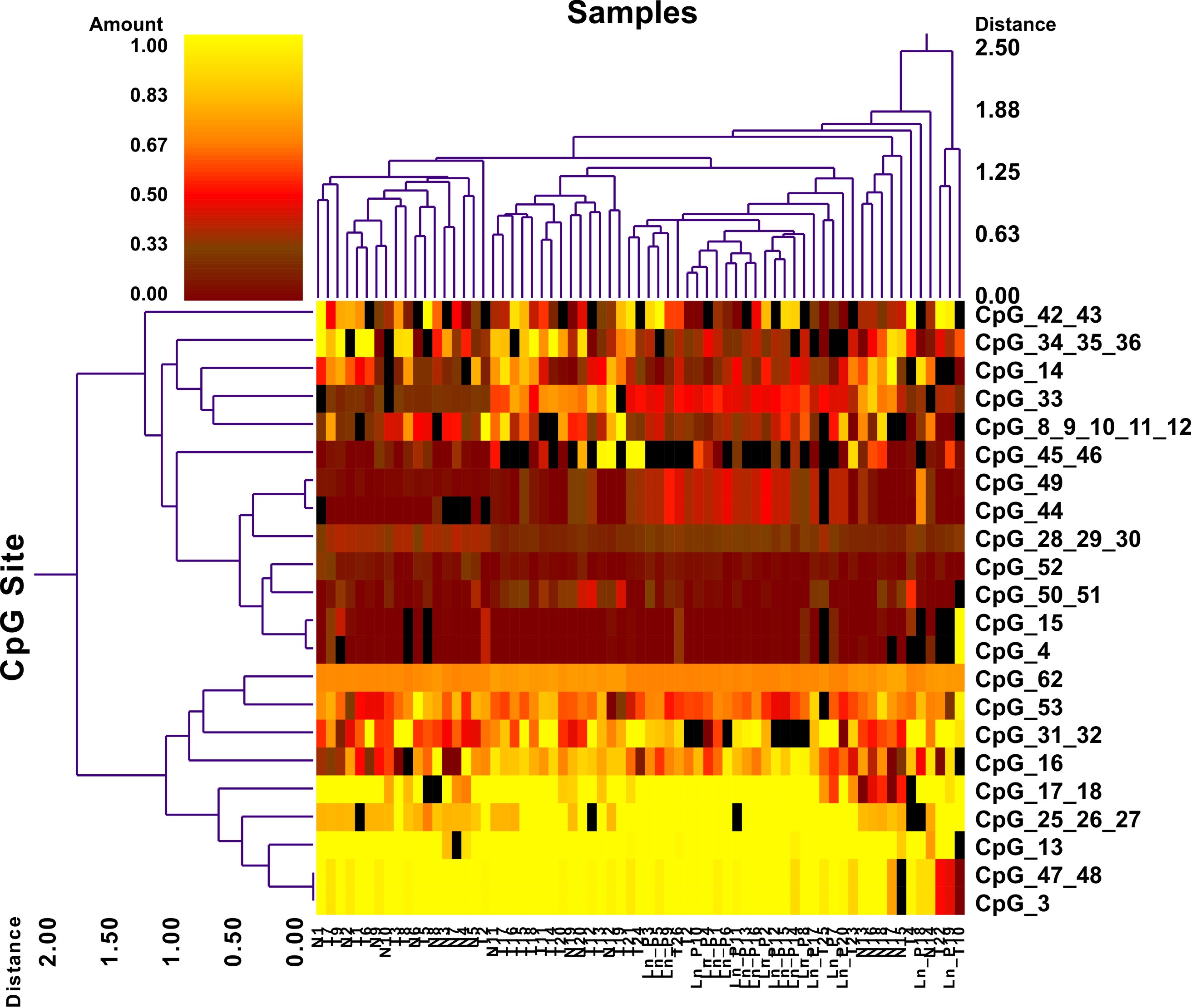


**Double dendrogram of *P16* gene:** Two-way hierarchical cluster analysis of breast cancer studied cohort. (Red clusters indicate 0% methylated, yellow clusters indicate 100% methylated, color gradient between red and yellow indicates methylation ranging from 0-100, and black clusters indicate not analyzed CpG sites).

***P21 (CDKN1A)* Gene**

| **Gene ID** | **Alternate gene name** | **locus** | **Function** | **Methylation effect on breast cancer** |
| --- | --- | --- | --- | --- |
| 1026 | Cyclin-dependent kinase inhibitor 1A (p21, Cip1) | 6p21.2 | Encodes protein that binds to and inhibits the activity of cyclin-CDK2 or -CDK4 complexes, and thus functions as a regulator of cell cycle progression at G1. This protein plays a regulatory role in S phase DNA replication and DNA damage repair. | direct |


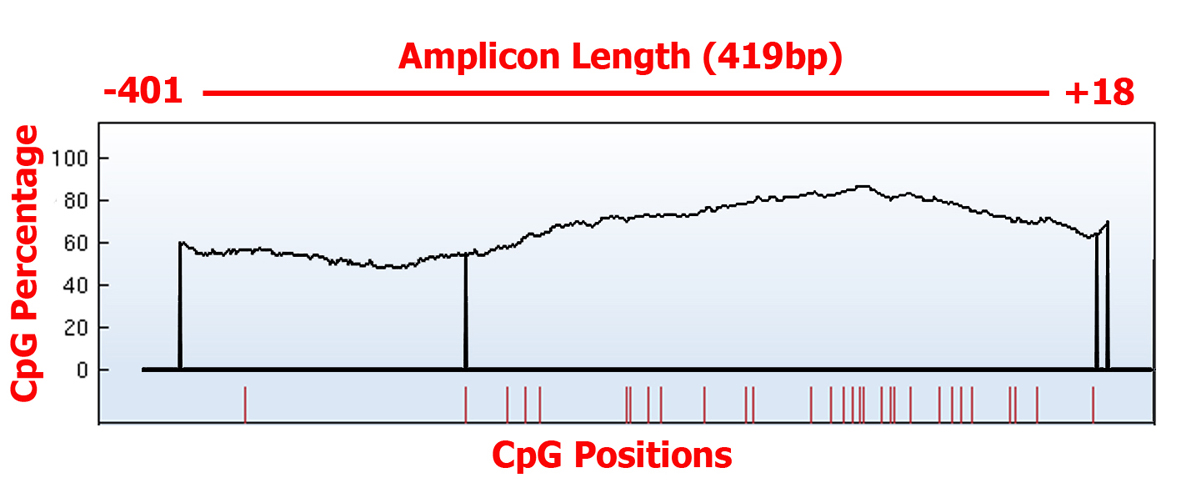


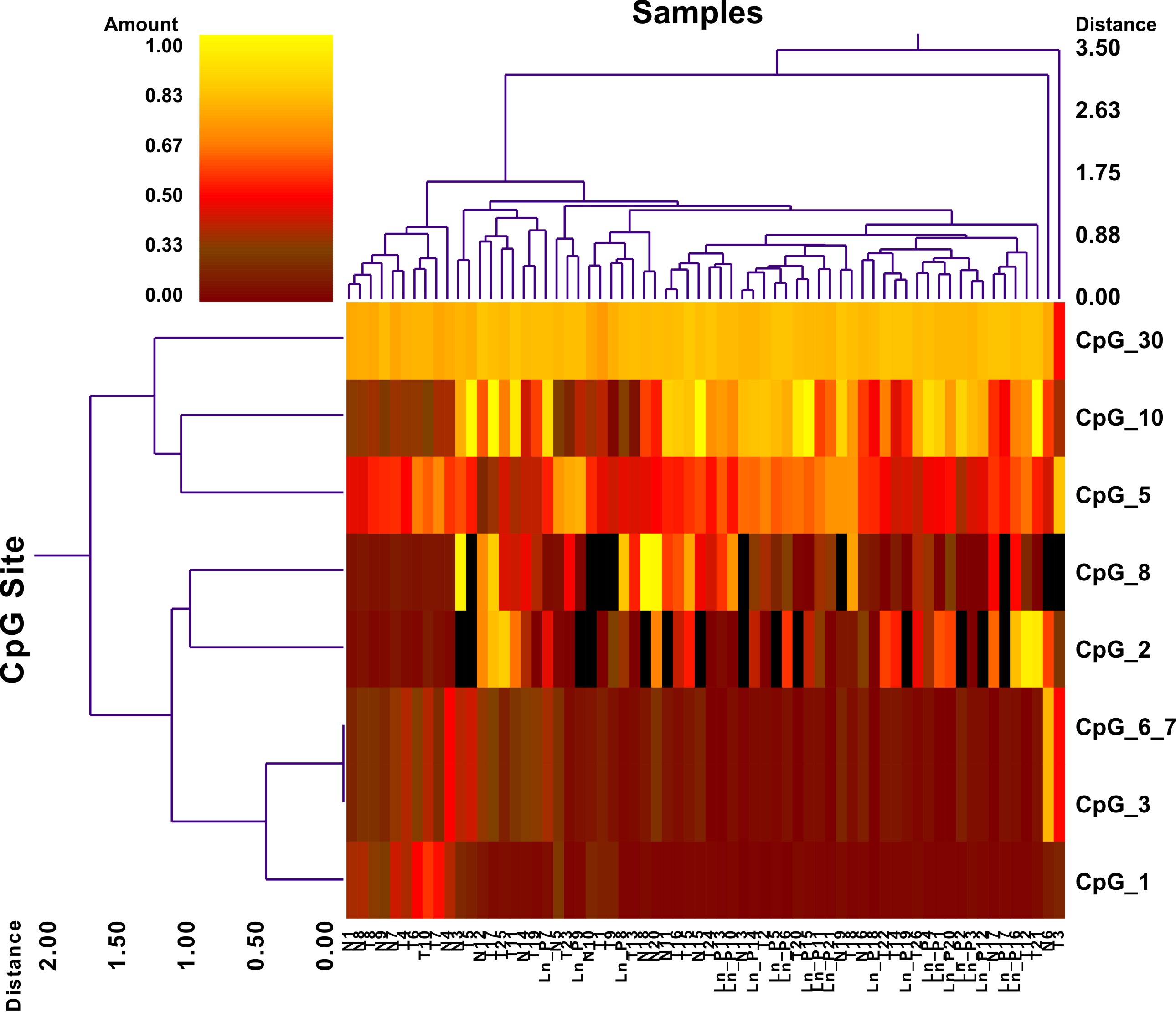


**Double dendrogram of *P21* gene:** Two-way hierarchical cluster analysis of breast cancer studied cohort. (Red clusters indicate 0% methylated, yellow clusters indicate 100% methylated, color gradient between red and yellow indicates methylation ranging from 0-100, and black clusters indicate not analyzed CpG sites).

***PTEN* Gene**

| **Gene ID** | **locus** | **Function** | **Methylation effect on breast cancer** |
| --- | --- | --- | --- |
| 5728 | 10q23.3 | This gene was identified as a tumor suppressor that is mutated in a large number of cancers at high frequency. The protein encoded this gene is a phosphatidylinositol-3,4,5-trisphosphate 3-phosphatase. | direct |


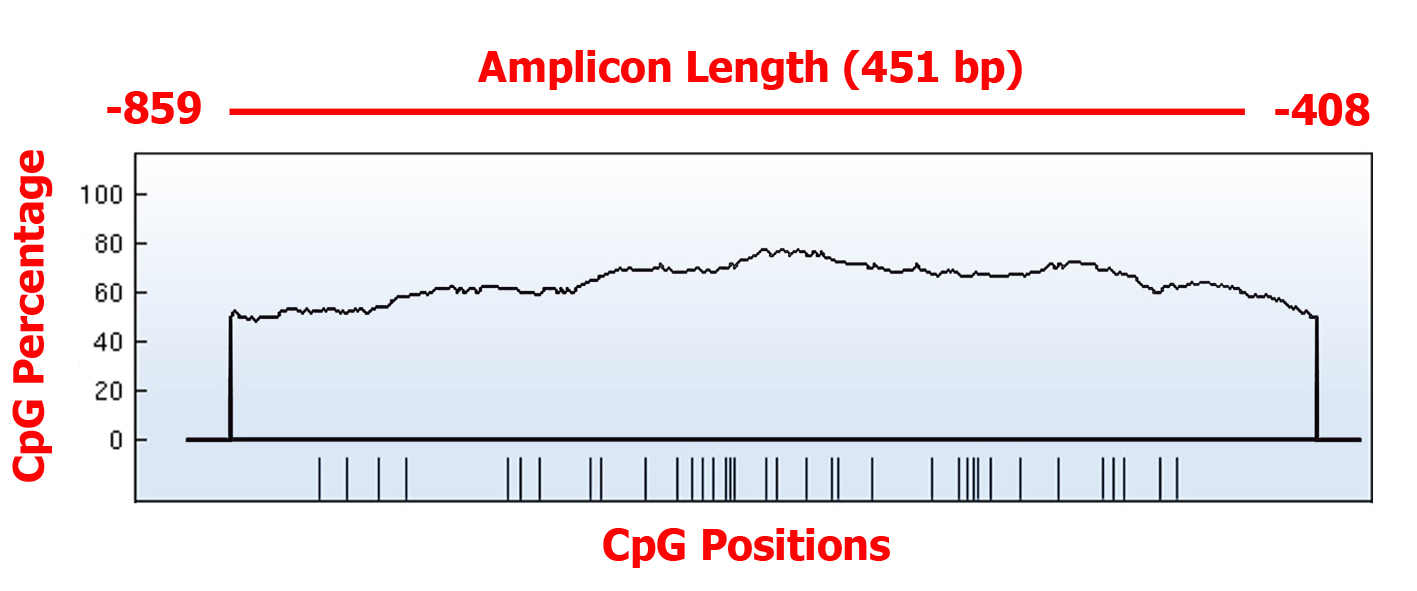


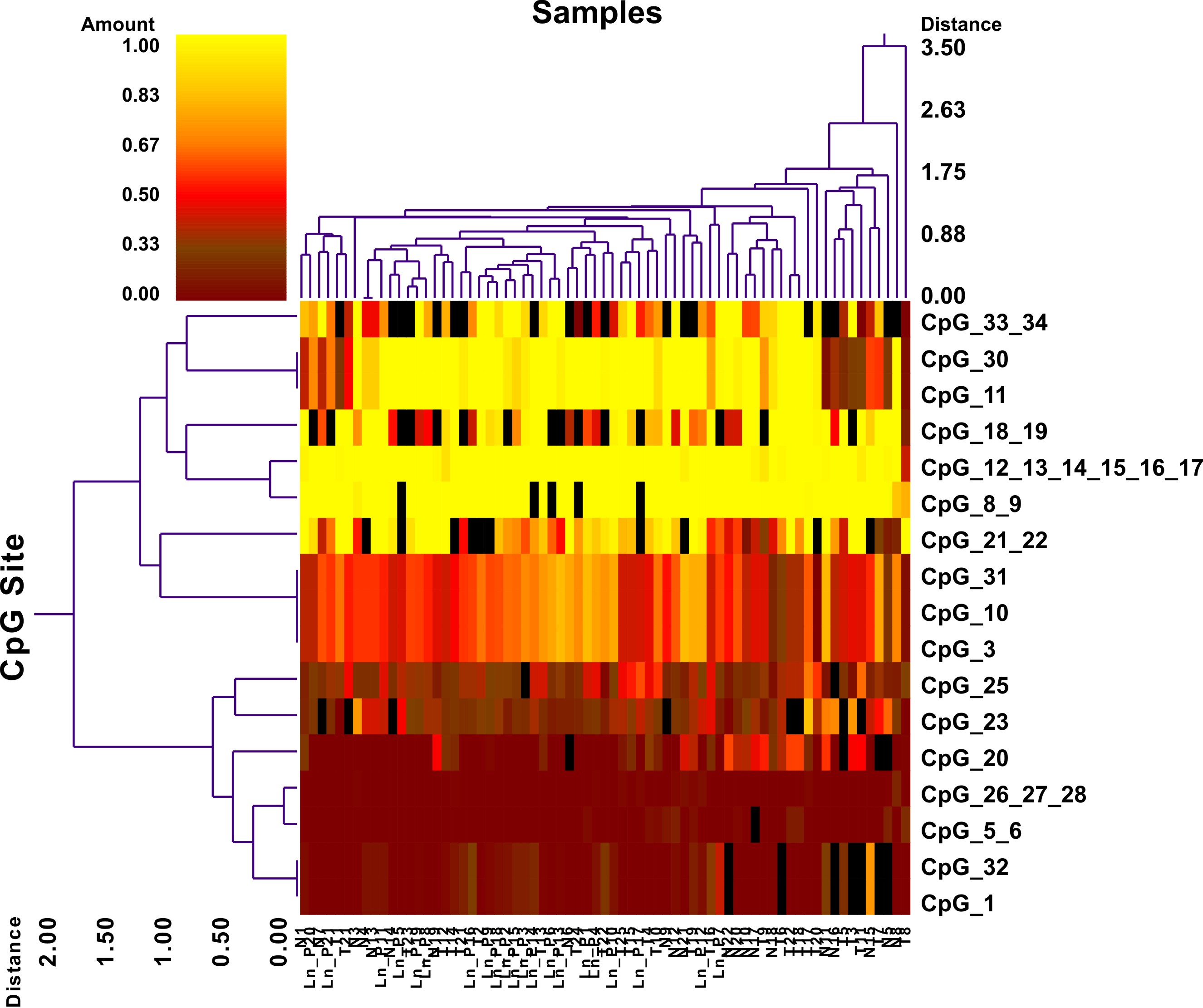


**Double dendrogram of *PTEN* gene:** Two-way hierarchical cluster analysis of breast cancer studied cohort. (Red clusters indicate 0% methylated, yellow clusters indicate 100% methylated, color gradient between red and yellow indicates methylation ranging from 0-100, and black clusters indicate not analyzed CpG sites).

***TIMP3* Gene**

| **Gene ID** | **Alternate gene name** | **locus** | **Function** | **Methylation effect on breast cancer** |
| --- | --- | --- | --- | --- |
| 7078 | Tissue inhibitor of metalloproteinase-3 | 22q12.3 | Suppresses tumour growth, angiogenesis, invasion and metastasis | direct |


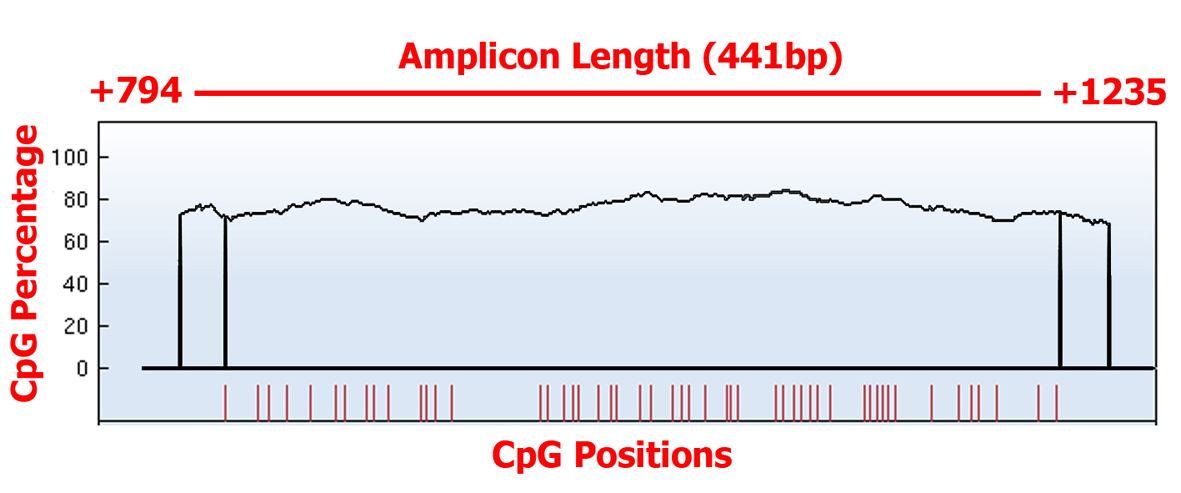


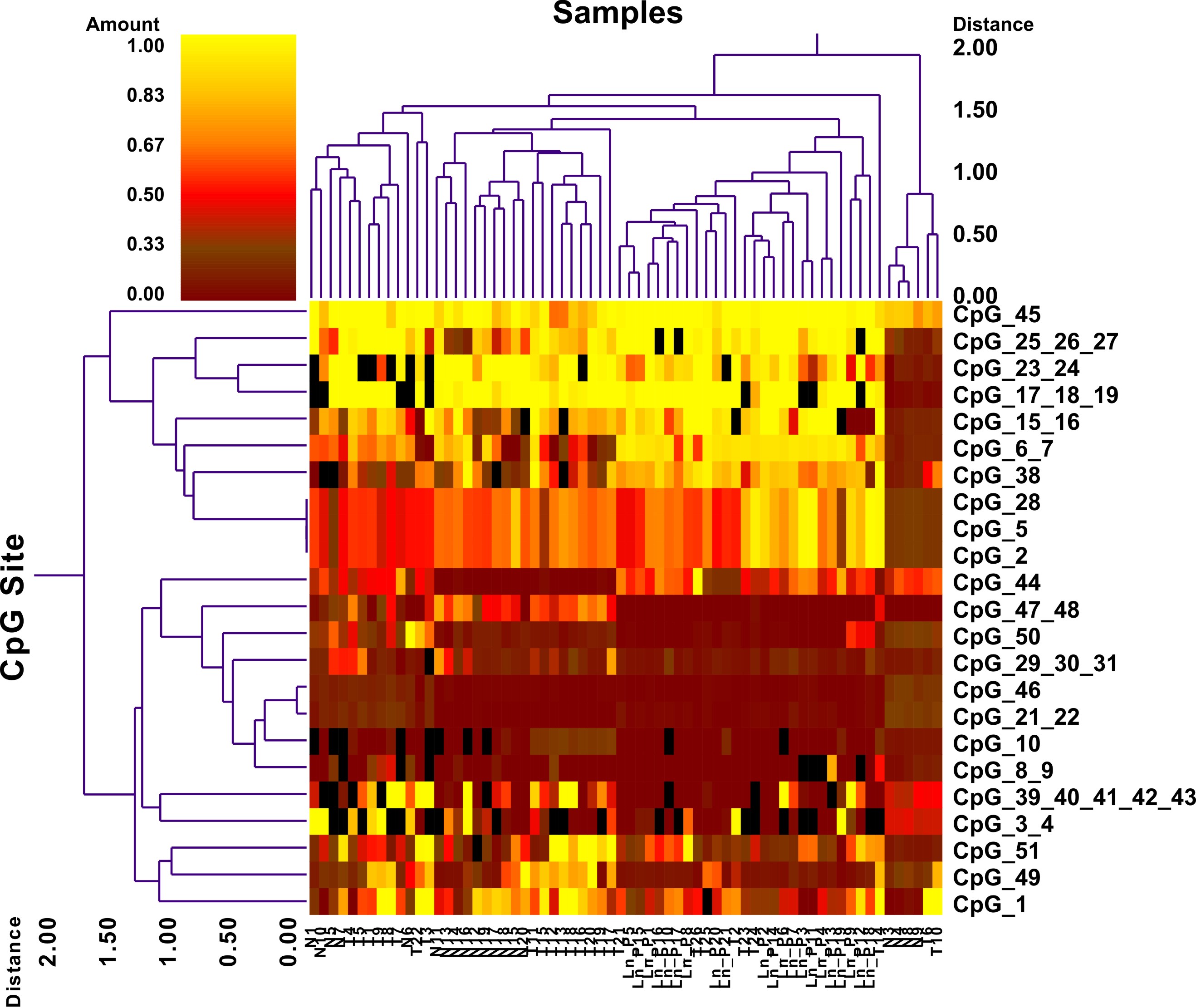


**Double dendrogram of *TIMP3* gene:** Two-way hierarchical cluster analysis of breast cancer studied cohort. (Red clusters indicate 0% methylated, yellow clusters indicate 100% methylated, color gradient between red and yellow indicates methylation ranging from 0-100, and black clusters indicate not analyzed CpG sites).
